# Unbalanced Expression of Glutathione Peroxidase 4 and Arachidonate 15-Lipoxygenase Affects Acrosome Reaction and In Vitro Fertilization

**DOI:** 10.3390/ijms23179907

**Published:** 2022-08-31

**Authors:** Mariana Soria-Tiedemann, Geert Michel, Iris Urban, Maceler Aldrovandi, Valerie B. O’Donnell, Sabine Stehling, Hartmut Kuhn, Astrid Borchert

**Affiliations:** 1 Department of Biochemistry, Charité—Universitätsmedizin Berlin, Corporate Member of Freie Universität Berlin and Humboldt-Universität zu Berlin, Charitéplatz 1, D-10117 Berlin, Germany; 2 Department of Transgenic Technologies, Charité—Universitätsmedizin Berlin, Corporate Member of Freie Universität Berlin and Humboldt-Universität zu Berlin, Lindenberger Weg 80, D-13125 Berlin, Germany; 3 Systems Immunity Research Institute, School of Medicine, Cardiff University, Cardiff CF14 4XN, UK; 4 Helmholtz Zentrum München, Institute of Metabolism and Cell Death, Ingolstädter Landstr. 1, D-85764 Neuherberg, Germany

**Keywords:** sperm, redox homeostasis, fertility, lipid peroxidation, eicosanoids

## Abstract

Glutathione peroxidase 4 (Gpx4) and arachidonic acid 15 lipoxygenase (Alox15) are counterplayers in oxidative lipid metabolism and both enzymes have been implicated in spermatogenesis. However, the roles of the two proteins in acrosomal exocytosis have not been explored in detail. Here we characterized Gpx4 distribution in mouse sperm and detected the enzyme not only in the midpiece of the resting sperm but also at the anterior region of the head, where the acrosome is localized. During sperm capacitation, Gpx4 translocated to the post-acrosomal compartment. Sperm from Gpx4^+/Sec46Ala^ mice heterozygously expressing a catalytically silent enzyme displayed an increased expression of phosphotyrosyl proteins, impaired acrosomal exocytosis after in vitro capacitation and were not suitable for in vitro fertilization. Alox15-deficient sperm showed normal acrosome reactions but when crossed into a Gpx4-deficient background spontaneous acrosomal exocytosis was observed during capacitation and these cells were even less suitable for in vitro fertilization. Taken together, our data indicate that heterozygous expression of a catalytically silent Gpx4 variant impairs acrosomal exocytosis and in vitro fertilization. Alox15 deficiency hardly impacted the acrosome reaction but when crossed into the Gpx4-deficient background spontaneous acrosomal exocytosis was induced. The detailed molecular mechanisms for the observed effects may be related to the compromised redox homeostasis.

## 1. Introduction

Fertilization is a complex process requiring a coordinated interaction between oocytes and sperm [1,2]. For proper oocyte fertilization, sperm carry specialized lysosome-like structures called acrosomes [3,4]. These organelles develop from the Golgi during spermatogenesis [4] and irregular acrosome formation (globozoospermia) leads to male infertility [5]. The acrosome covers the anterior half of the head of mammalian sperm. Within the head, the acrosome is located between the plasma membrane and the nucleus, and thus, it is not directly exposed to the cell surface [6]. When the plasma membrane fuses with the outer acrosomal membrane, the content [7,8,9] of the apical acrosome is liberated and this process is called acrosome reaction [10]. The released hydrolytic enzymes break down constituents of the zona pellucida protecting the plasma membrane of the oocyte [11]. Consequently, the acrosome reaction enables penetration of the haploid nucleus of the sperm into the oocyte cytoplasm forming the male pro-nucleus of the pre-embryo. To be able to fertilize the egg, sperm undergo a series of functional changes in the female reproductive tract known as capacitation [12]. This process is characterized by a series of phosphorylation events, modifications to the plasma membrane, cholesterol efflux, and hyperactivation. Only capacitated spermatozoa can undergo the acrosomal reaction [13]. One of the best-characterized events in sperm capacitation is an increase in protein phosphorylation of tyrosine residues [14,15,16].

Redox reactions play an important role in gametogenesis, fertilization, and embryonic development [17,18,19]. A number of pro- [20,21,22] as well as anti-oxidative enzymes [23,24] have been implicated in the redox homeostasis of sperm. Two of these enzymes [21,25] are glutathione peroxidase 4 (Gpx4) and arachidonic acid 15-lipoxygenase (Alox15) and functional inactivation of the corresponding genes induces male subfertility [21,26,27]. The two enzymes constitute counterplayers in the metabolism of hydroperoxy lipids [28,29], and thus, their balanced expression is important for intracellular redox homeostasis. ALOX15 orthologs oxygenate complex lipids carrying polyunsaturated fatty acids to the corresponding hydroperoxides and these hydroperoxy lipids may induce secondary oxidation reactions [30,31]. GPX4 orthologs reduce such complex hydroperoxy lipids to the corresponding hydroxy derivatives at the expense of reduced glutathione, and thus, this enzyme lowers the cellular oxidation potential [32,33].

ALOX15 orthologs are expressed in testes of various mammals and have been implicated in spermatogenesis [34,35,36] and in the acrosome reaction [37,38]. On the other hand, *Alox15^−/−^*, *Alox12^−/−^*, and *Alox5^−/−^* knockout mice develop normally and do not show major reproductive defects [39,40,41]. However, more detailed studies on the reproductive behavior of *Alox15^−/−^* mice suggested that male individuals are subfertile [21,27]. The underlying molecular mechanisms remain unclear but it has been suggested that epididymal sperm maturation, in particular, the movement of the cytoplasmic droplet containing remnants of spermatogenic organelles as well as Alox15 [20] is reduced in *Alox15^−/−^* sperm [21].

Gpx4 is also important for sperm development [24,25,27]. The enzyme is high-level expressed in mammalian testes [25,42,43] and specific polymorphisms in the human *GPX4* gene correlate with male infertility [44]. In sperm, the GPX4 protein exhibits dual functionality. It works as catalytically active peroxidase but also functions as a structural protein for the formation of the mitochondrial capsule and for proper paternal chromatin decondensation during fertilization [24,45]. *Gpx4*^−/−^ mice die early during embryogenesis [46,47] and similar results were obtained for homozygous knock-in mice expressing a catalytically silent Gpx4^Sec46Ala/Sec46Ala^ mutant [26,48]. Thus, spermatogenesis could not be studied in these animals. To overcome this problem, spermatocyte-specific conditional knockout mice were created. These mice are infertile, their testes display decreased numbers of spermatozoa and isolated epididymal sperm were unable to fertilize oocytes in vitro [49]. The sperm of these animals showed severe structural abnormalities and exhibit reduced forward motility [49].

Taken together, these data suggest roles for Alox15 and Gpx4 in sperm development. However, little is known about the functionality of the two enzymes in oocyte fertilization, in particular in the acrosome reaction [1]. This study aimed at exploring in more detail the potential roles of Gpx4 and Alox15 in acrosomal exocytosis and in vitro fertilization. To achieve this goal, we first explored the expression of the two enzymes in mouse sperm and followed their subcellular distribution during in vitro capacitation and acrosomal exocytosis. Then we employed *Gpx4^+/Sec46Ala^* knock-in mice (Gpx4^+/U46A^), which heterozygously express a catalytically silent Gpx4 mutant [48], systemic *Alox15*-deficient (*Alox15^−/−^*) animals [39], and mice carrying both genetic alterations (Gpx4^+/U46A^ + *Alox15^−/−^*) [27], to compare the extent of the in vitro acrosome reaction with sperm prepared from wild-type mice. We found that Gpx4 is abundantly present in the sperm head of wild-type sperm and that its subcellular distribution is altered during sperm capacitation. Gpx4^+/U46A^ sperm exhibit a reduced capacity for acrosomal exocytosis associated with increased protein tyrosine phosphorylation. When *Alox15* deficiency was crossed into this genetic background spontaneous acrosome reaction was induced during the capacitation period. Thus, in addition to their functions in spermatogenesis Alox15 and Gpx4 may also play a role in the acrosome reaction.

## 2. Results

### 2.1. Capacitation Prepares Isolated Cauda Sperm for the Acrosome Reaction

**Figure 1:**
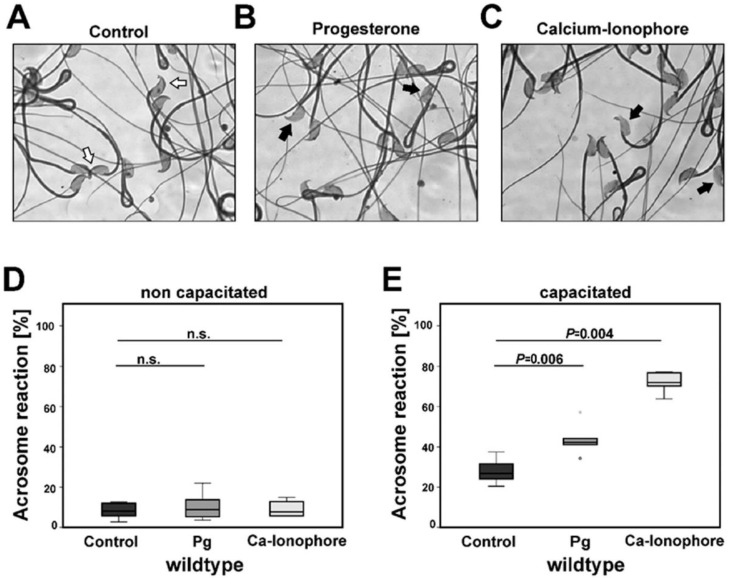
In vitro induction of the acrosome reaction of sperm prepared from wild-type mice. Sperm were prepared from wild-type mice (C57BL/6N). After capacitation acrosome reaction was induced by the addition of progesterone or calcium ionophore A23187 as described in the “Materials and Methods” section. (**A**) Staining of capacitated wild-type sperm. The white arrows indicate the acrosome structure. (**B**) Staining of sperm for which the acrosome reaction was initiated by progesterone. Black arrows point toward sperm that have lost their acrosomes. (**C**) Staining of sperm in which the acrosome reaction was initiated by A23187. Black arrows point toward sperm that have lost their acrosomes. 50-fold magnification. (**D**,**E**) Quantification of the acrosome reaction of non-capacitated (**D**) and capacitated (**E**) sperm. Significances were calculated using the Mann–Whitney U test. The experiment was carried out with six different individuals (*n* = 6). The percentages of capacitated and non-capacitated acrosome reacted spermatozoa were calculated. *p*-values < 0.05 were considered statistically significant. n.s. not significant.

From Figure 1A it can be seen that epididymal cauda sperm show a regular structure with a head, midpiece, and tail. On top of the head carrying the nucleus, the optically dense acrosomes are visible.

After capacitation and the subsequent induction of the acrosome reaction with progesterone (Figure 1B) and calcium ionophore (Figure 1C), the acrosomes are lost in many cells. To judge the intensity of the acrosome reaction, we quantified the percentage of acrosome-free sperm. When this readout parameter was determined for non-capacitated sperm of wild-type mice (C57BL/6N), a low basal acrosome reaction (2–20%) was observed (Figure 1D). However, when these compounds were added to capacitated sperm, the relative numbers of acrosome-free sperm were significantly increased and in the presence of calcium ionophore, more than 70% of the sperm had lost their acrosomes (Figure 1E).

### 2.2. Subcellular Distribution of Gpx4 and Alox15 in Non-Capacitated and Capacitated Sperm

**Figure 2:**
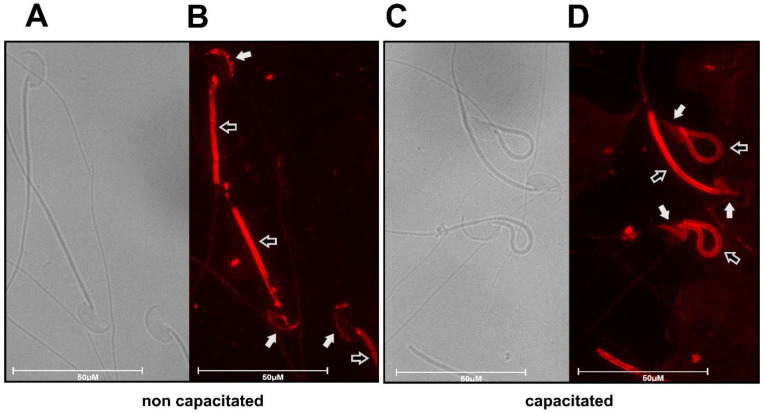
Immunohistochemical Gpx4 staining of wild-type mouse sperm before and after the capacitation. Sperm from epididymal cauda and vas deferens were extracted and capacitated. Immunohistochemical staining was carried out as described in the “Materials and Methods” section. (**A**) Phase contrast visualization of non-capacitated sperm, (**B**) immunohistochemical Gpx4 staining of non-capacitated sperm, 100-fold. Midpiece (white framed arrows) and head (white arrows) were stained Gpx4 positive. 87% of sperm exhibited Gpx4-positive staining of the head. (**C**) Phase contrast visualization of capacitated sperm, (**D**) immunohistochemical Gpx4 staining of capacitated sperm, 100-fold. Midpiece (white framed arrows) and the post-acrosomal region (white arrows) were stained Gpx4 positive, whereas the anterior region of the sperm head was devoid of Gpx4. 73% of sperm exhibited Gpx4 positive staining of the post-acrosomal region.

To explore the role of Gpx4 and Alox15 in the acrosome reaction, we first determined the subcellular distribution of the two proteins in non-permeabilized, non-capacitated sperm extracted from the cauda epididymis and the ductus deferens. We detected abundant Gpx4 expression in the midpiece of the sperm (Figure 2B).

However, we also observed strong immunostaining in the anterior region of the sperm head (Figure 2B and Figure S10B), and these data suggested the presence of Gpx4 at the apical side of the sperm head, covering the acrosome. Next, we explored whether the subcellular distribution of Gpx4 was altered during capacitation and acrosome reaction. As indicated in Figure 2D and Figure S10D, the staining signal at the apical side of the sperm head disappeared during capacitation. Instead, Gpx4 was detected in the post-acrosomal segment of the sperm head. In contrast, when the acrosome reaction was induced by progesterone or calcium ionophore, the subcellular distribution of Gpx4 was not altered any further. As for capacitated sperm, we detected abundant Gpx4 staining in the midpiece and the post-acrosomal segment of the headpiece. Next, we performed immunohistochemical staining with a polyclonal antibody directed against pure rabbit ALOX15 which strongly cross-reacts with native and recombinant mouse Alox15. However, we were unable to reliably detect the expression of Alox15 in mouse cauda sperm.

### 2.3. Alox15 Products Are Present in the Cellular Lipids of Mouse Sperm

**Figure 3:**
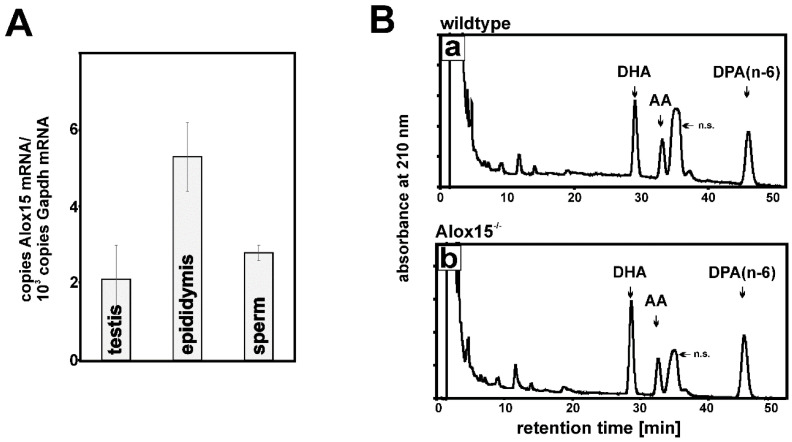
Expression of Alox15 in mouse wild-type sperm and characterization of the PUFA composition of sperm of different genotypes. (**A**) Quantitative RT-PCR of Alox15 in the testis, epididymis, and sperm of wild-type mice. Total RNA preparation and qRT-PCR were carried out as described in “Materials and Methods”. The Alox15 mRNA copy number/10^3^ GAPDH mRNA copy number ratio was calculated to quantify the Alox15 expression level. (**B**_**a**) RP-HPLC chromatogram of the hydrolyzed lipid extracts of wild-type sperm. Lipid extraction, alkaline hydrolysis, and HPLC analyses were carried out as described in the “Materials and Methods” section. PUFA peaks were identified by co-chromatography with commercial standards. (**B**_**b**) RP-HPLC chromatogram of the hydrolyzed lipid extracts of *Alox15^−/−^* sperm. N = 12 animals of each genotype were analyzed. Statistical evaluation showed no significant differences in the relative content of the analyzed main PUFAs of sperm (Figure S2). DHA: docosahexaenoic acid, AA: arachidonic acid, DPA: docosapentaenoic acid, n.s.: no specific fatty acid.

To obtain independent evidence of the activity of Alox15 in sperm we employed a dual research strategy:

(i) We quantified by qRT-PCR the steady-state concentrations of Alox15 mRNA in the testis, epididymis, and in extracted sperm. Here we found (Figure 3A) that Alox15 mRNA could be detected in all these samples but the expression levels were rather low (2.8 ± 0.2 Alox15 mRNA copies/10^3^ GAPDH mRNA copies for cauda sperm).

**Figure 4:**
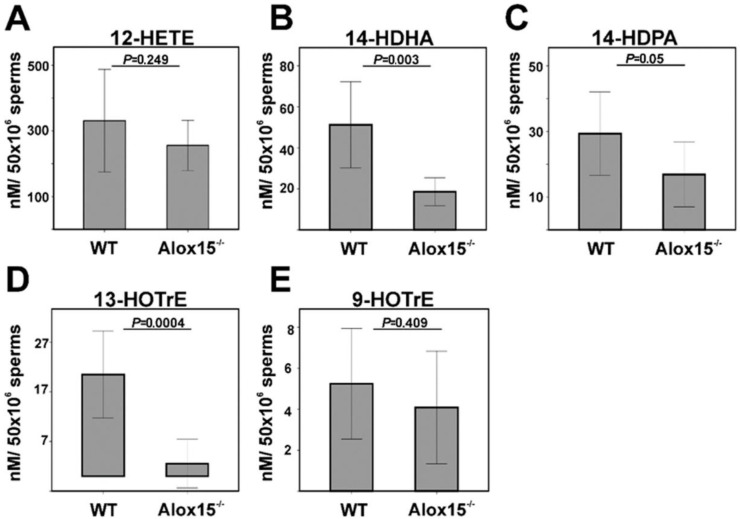
Quantification of Alox15 products in the lipid extract of sperm prepared from wild-type mice and Alox15^−/−^ animals. Epididymal cauda sperm were prepared from wild-type mice and Alox15-deficient animals, their total lipids were extracted, hydrolyzed under alkaline conditions, and selected hydroxy PUFAs were quantified by LC-MS/MS (see “Materials and Methods”). (**A**) 12-hydroxy arachidonic acid (12-HETE), (**B**) 14-hydroxy docosahexaenoic acid (14-HDHA), (**C**) 14-hydroxy-docosapentaenoic acid (14-HDPA), (**D**) 13-hydroxy alpha linolenic acid (13-HOTrE), and (**E**) 9-hydroxy alpha linoleic (9-HOTrE). The degree of significance (*p*-values) is given above the columns. Significances were calculated using Student’s *t*-test. N = 8 animals from each genotype were analyzed and *p*-values < 0.05 were considered statistically significant.

Similarly, a low expression level of Alox15 was quantified in Gpx4^+/U46A^ sperm [3.0 ± 0.3 Alox15 mRNA copies/10^3^ GAPDH mRNA copies for cauda sperm].

(ii) Next, we attempted to detect Alox15 products in the sperm lipids. To specifically look for such products we first quantified the polyunsaturated fatty acid pattern in the sperm lipids. Here we found (Figure 3B_a) that docosahexaenoic acid (DHA), arachidonic acid (AA), and n-6 docosapentaenoic acid (DPA) were the dominant polyenoic fatty acids in wild-type mouse sperm. Similar chromatograms were observed for Alox15^−/−^ sperm (Figure 3B_b). These PUFAs were identified by co-chromatography with authentic standards (co-injections) and the retention times of 10 different PUFA standards were determined (Figure S1). The three major sperm PUFAs constitute suitable substrates for Alox15. If this enzyme is catalytically active in these cells, the steady-state concentrations of Alox15 products derived from these PUFAs should be higher in wild-type mice when compared with Alox15^−/−^ animals. Mouse Alox15 converts arachidonic acid predominantly to 12-HpETE, which is rapidly reduced to 12-HETE via Gpx-dependent reactions [50]. When we quantified the 12-HETE content in the hydrolyzed lipid extracts of wild-type and Alox15^−/−^ sperm, we did not see significant differences (Figure 4A).

Thus, this readout parameter did not provide major evidence for the catalytic activity of Alox15 in mouse sperm. It should, however, be stressed that 12-HETE is not only formed by mouse Alox15 but also by mouse Alox12 (platelet isoform) and mouse Alox12b (skin isoform). When we analyzed the expression of Alox12 in sperm by qRT-PCR we only detected very low expression levels when compared with Alox15 (Figure S3). In contrast, in the testis and epididymis, the Alox12 mRNA expression was about 10-fold higher than that of Alox15. These data provide a plausible explanation for our finding that Alox15 deficiency did not reduce endogenous 12-HETE formation. When mouse recombinant Alox15 oxygenates DHA in vitro, 14-HDHA is the dominant product [51]. Thus, we quantified 14-HDHA in the hydrolyzed lipid extracts and found that this lipid was present in significantly (*p* = 0.003) lower quantities in Alox15^−/−^ sperm (Figure 4B). A representative mass spectrum of 14-HDHA detected in the hydrolyzed lipid extracts of wild-type sperm is shown in Figure S6. Similar significant differences (Figure 4C,D) were observed for Alox15 products formed from DPA (14-HDPA, *p* = 0.05) and ALA (13-HOTrE, *p* = 0.0004). In control measurements, no significant differences were observed for 9-HOTrE (Figure 4E), which is not formed by Alox15.

### 2.4. Heterozygous Expression of the Catalytically Inactive Sec46Ala Gpx4 Mutant Impairs Acrosomal Exocytosis

**Figure 5:**
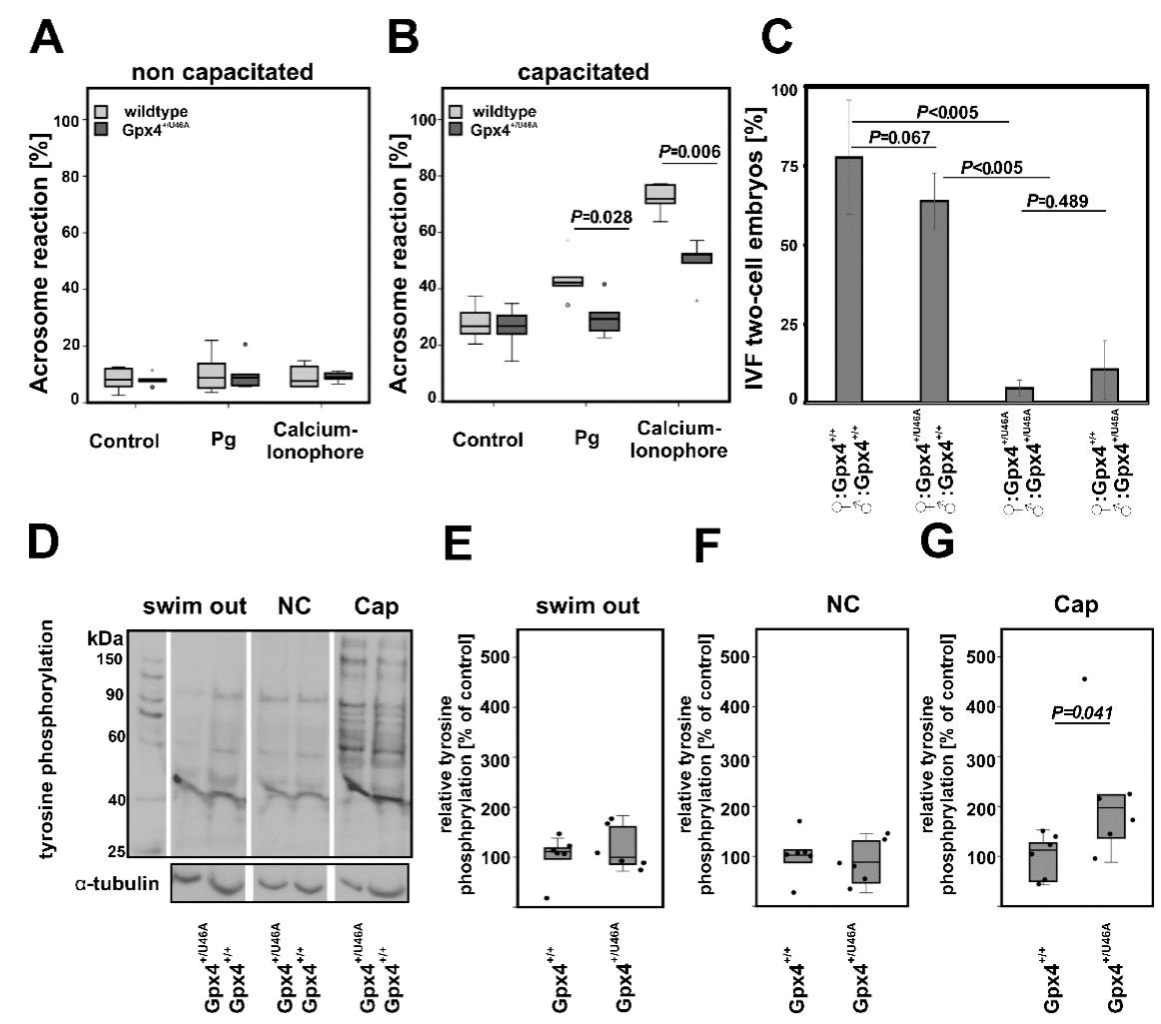
Heterozygous expression of catalytically inactive Gpx4 impairs the capability of the acrosome reaction and reduces the rate of in vitro fertilization. (**A**,**B**) In vitro acrosome reaction. Epididymal cauda sperm were extracted from wild-type mice and Gpx4^+/U46A^ animals. The acrosome reaction was induced in non-capacitated (**A**) and capacitated (**B**) sperm of the same sperm preparation with progesterone and calcium ionophore A23187. The extent of the acrosome reaction was quantified as described in “Materials and Methods”. The control panels show the degree of spontaneous acrosomal reaction in solvent-treated samples. Significances were calculated using the Mann–Whitney U test. The experiment was carried out with 5–6 different individuals. At least 200–300 stained cells per treatment were scored using an Axioskop microscope (Zeiss, Jena, Germany) with a 40× objective clear field. Heads of sperm with the acrosome were stained dark blue (intact, not reacted acrosome) and without acrosome unstained (reacted acrosome). The percentage of capacitated and non-capacitated acrosome-reacted spermatozoa was calculated. *p*-values < 0.05 were considered statistically significant. (**C**) In vitro fertilization of wild-type (Gpx4^+/+^) and Gpx4^+/U46A^ oocytes with sperm prepared from wild-type mice (Gpx4^+/+^) and Gpx4^+/U46A^ animals. Cumulus-oocyte complexes (COCs) were isolated from stimulated (see “Materials and Methods”) female mice (Gpx4^+/+^ and Gpx4^+/U46A^) and were fertilized in vitro with wild-type sperm (columns 1, 2) and with sperm prepared from U46A-Gpx4^+/−^ mice (columns 3, 4) as described in “Materials and Methods”. After a 28 h incubation period, the relative shares of 2-cell embryos were quantified. The fertilization rates were calculated as the total number of 2-cell embryos divided by the total number of oocytes ×100. (**D**) Level of protein tyrosine phosphorylation during in vitro capacitation in sperm prepared from wild-type mice (Gpx4^+/+^) and Gpx4^+/U46A^ animals. Total protein tyrosine phosphorylation from protein extracts obtained from (**E**) freshly isolated (swim-out), (**F**) non-capacitated (NC), and (**G**) capacitated (Cap) spermatozoa as described in “Materials and Methods”. The intensities of the total protein tyrosine phosphorylation levels under the appropriate conditions were quantified using Image J and normalized to the respective intensity of alpha tubulin. The normalized values were further related to the corresponding control mice (Gpx4^+/+^). Significances were calculated using the Mann–Whitney U test. The experiment was carried out with six different individuals of each genotype.

Our immunohistochemical staining indicated that Gpx4 is expressed in the anterior region of the sperm head of normal sperm and that its subcellular localization is altered during capacitation (Figure 2B,D). To test this hypothesis, we employed Gpx4^+/U46A^ mice, extracted cauda and ductus deferens sperm, and quantified the extent of the in vitro acrosome reaction. For this purpose, the extracted sperm were capacitated and then stimulated with either progesterone or calcium ionophore to induce the acrosome reaction. When this reaction was quantified for non-capacitated sperm of wild-type and Gpx4^+/U46A^ mice, no significant differences were observed regardless of whether progesterone or calcium ionophore was used as an inducer of acrosomal exocytosis (Figure 5A). After the capacitation of spermatozoa, no significant differences were seen when sperm of the two genotypes were treated with DMSO as a solvent control (Figure 5B). However, when the acrosome reaction was initiated with progesterone, no additional acrosomal exocytosis was found in Gpx4^+U46A^ mice when compared with wild-type controls. In contrast, the intensity of the acrosome reaction nearly doubled for wild-type sperm (*p* = 0.028). When calcium ionophore was used as an inducer, the difference between the two genotypes was statistically even more significant (*p* = 0.006).

### 2.5. Homozygous Expression of Catalytically Active Gpx4 in Sperm Is Essential for In Vitro Fertilization

Since the intensity of the in vitro acrosome reaction is reduced in Gpx4^+/U46A^ sperm, it was expected that these cells would not be suitable for in vitro fertilization. When wild-type (Gpx4^+/+^) cumulus-oocytes-complexes (COCs) were incubated in vitro with wild-type sperm (Gpx4^+/+^) under our experimental conditions, we usually observed that about 60–90% of oocytes developed into the 2-cell embryo stage. When Gpx4^+/U46A^ oocytes were incubated with wild-type sperm, we also observed that more than 60% of the oocytes developed into the 2-cell stage (Figure 5C). However, when Gpx4^+/U46A^ oocytes and Gpx4^+/U46A^ sperm were used for in vitro fertilization, only about 5% of the oocytes developed into 2-cell embryos. Similarly, low success rates were found when Gpx4^+/+^ oocytes and Gpx4^+/U46A^ sperm were used for in vitro fertilization. Thus, in vitro fertilization was more than 10-fold less efficient when Gpx4^+/U46A^ sperm were employed.

### 2.6. Heterozygous Expression of Catalytically Inactive Gpx4 in Sperm Increases Tyrosine Phosphorylation during In Vitro Capacitation

The capacitation process involves an increase in global tyrosine phosphorylation [14] and is modulated by the redox status of cells [52,53]. To explore whether the impaired in vitro acrosome reaction in Gpx4^+/U46A^ spermatozoa was caused by perturbations in the capacitation process, we compared the degree of protein tyrosine phosphorylation after the capacitation of Gpx4^+/U46A^ sperm and Gpx4^+/+^ control cells (Figure 5D–G). For this purpose, total sperm proteins were extracted and evaluated by Western blot analysis using an anti-phosphotyrosine antibody. It can be seen that sperm capacitation increased the number of phosphorylated proteins and the band intensities (Figure 5D, Cap). Densitometric quantification of the immunoblots suggested that the degree of protein phosphorylation was not significantly different between wild-type mice (Gpx4^+/+^) and Gpx4^+/U46A^ animals when swim-out sperm (Figure 5E) and non-capacitated sperm (Figure 5F) were compared. In contrast, capacitated sperm of Gpx4^+/U46A^ animals exhibited a significantly increased degree of protein phosphorylation (Figure 5G).

### 2.7. Expression of Catalytically Inactive Gpx4^+/U46A^ Mutant and Additional Alox15 Deficiency Induces Spontaneous Acrosome Reaction

**Figure 6:**
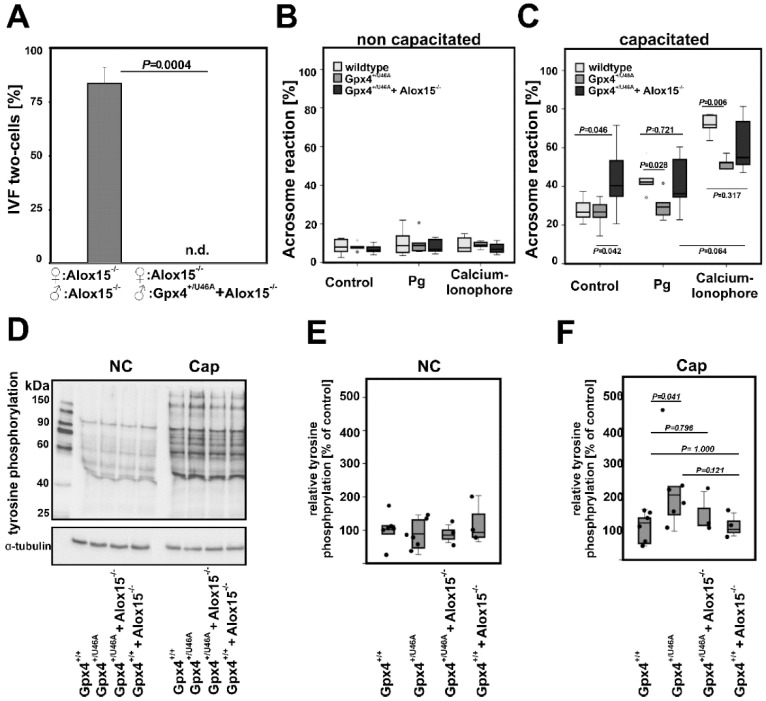
Impact of sperm Alox15 deficiency on the success rate of in vitro fertilization and the extent of the acrosome reaction. (**A**) In vitro fertilization of Alox15-deficient oocytes (Alox15^−/−^) with sperm prepared from Alox15^−/−^ mice (left column) and Alox15^−/−^ + Gpx4^+/U46A^ (right column) animals. Cumulus-oocyte complexes (COCs) were isolated from female Alox15^−/−^ mice and were fertilized in vitro with sperm extracted from males of the same genotype (left column). Next, we employed the same oocytes and attempted to fertilize them in vitro with sperm prepared from Gpx4^+/U46A^ + Alox15^−/−^ mice. Here we observed complete prevention of in vitro fertilization. The fertilization rates were calculated as the total number of 2-cell embryos divided by the total number of oocytes ×100. This experiment was carried out when the corresponding mouse strains were prepared for moving to another animal facility within Charité. Moreover, data are consistent with the outcome of other in vitro fertilization experiments performed when oocytes of other genetically modified mouse strains were crossed with Gpx4^+/U46A^ sperm. In all these experimental setups, Gpx4^+/U46A^ sperm were not suitable for in vitro fertilization regardless of the genotypes of several other gene loci. For these crossings, alternative in vitro fertilization strategies were employed. (**B**,**C**) In vitro acrosome reaction. Epididymal cauda sperm were extracted from wild-type mice, Gpx4^+/U46A^ animals, and Gpx4^+/U46A^ + Alox15^−/−^ mice. The acrosome reaction was induced in non-capacitated (**B**) and capacitated (**C**) sperm with progesterone and A23187. The percentage of the acrosome reaction was quantified as described in “Materials and Methods”. Control indicates the spontaneous acrosomal reaction in the DMSO solvent control sample. Significances were calculated using the Mann–Whitney U test. The experiment was carried out with 5–6 different individuals. At least 200–300 stained cells per treatment were scored using Axioskop microscope (Zeiss, Jena, Germany) with a 40-fold objective clear field. Heads of sperm with the acrosome were stained dark blue (intact, not reacted acrosome) and without acrosome (unstained, reacted acrosome). The percentage of capacitated and non-capacitated acrosome-reacted spermatozoa was calculated. *p*-values < 0.05 were considered statistically significant. (**D**) Level of protein tyrosine phosphorylation during in vitro capacitation in sperm prepared from wild-type (Gpx4^+/+^), Gpx4^+/U46A^, *Gpx4^+/U46A^* + *Alox15^−/−^*, and *Gpx4^+/+^* + *Alox15^−/−^* animals. Total protein tyrosine phosphorylation from protein extracts obtained from (**E**) non-capacitated (NC) and (**F**) capacitated (Cap) spermatozoa as described in “Materials and Methods”. The intensities of the total protein tyrosine phosphorylation levels under the appropriate conditions were quantified using Image J and normalized to the respective intensity of alpha tubulin. The normalized values were further related to the corresponding control mice (Gpx4^+/+^). Significances were calculated using the Mann–Whitney U test. The experiments were carried out with 3–6 different individuals of each genotype. *p*-values < 0.05 were considered statistically significant.

Complete Alox15 deficiency in sperm and oocytes neither impacted the intensity of the in vitro acrosome reaction (Figure S4) nor the success rate of in vitro fertilization (Figure 6A).

In fact, when we carried out in vitro fertilization employing Alox15^−/−^ sperm and Alox15^−/−^ oocytes, we calculated a fertilization rate of more than 80% (Figure 6A). However, when we employed *Alox15^−/−^* oocytes and incubated them with *Gpx4^+/U46A^* + *Alox15^−/−^* sperm, we did not see any 2-cell embryos. These data suggest that functional Gpx4-deficient sperm may not be suitable for in vitro fertilization.

To explore the molecular basis for this finding we reasoned that a defective acrosome reaction of *Gpx4^+/U46A^* + *Alox15^−/−^* sperm may be responsible for this observation. To test this hypothesis, we quantified the intensity of the acrosome reaction using sperm from different genotypes. Usually, effective acrosomal exocytosis requires the capacitation process, which prepares the sperm for the subsequent acrosome reaction. However, a small share of sperm (usually around 10%) undergo premature acrosomal exocytosis during the capacitation period and this process is termed spontaneous acrosome reaction. When we quantified the number of post-acrosomal sperm (cells that have lost the acrosome) among non-capacitated sperm prepared from wild-type, Gpx4^+/U46A,^ and Gpx4^+/U46A^ + Alox15^−/−^ mice (Figure 6B), we only quantified relatively small shares (<10%) and did not detect significant differences between the different genotypes. In contrast, different results were obtained for capacitated sperm (Figure 6C) and here a strong impact of the genotype was observed. Capacitated sperm of wild-type mice and Gpx4^+/U46A^ animals showed a subtle increase in the spontaneous acrosome reaction (compare the control lanes of Figure 6B,C) but there was no significant difference between the two genotypes. In contrast, the share of post-acrosomal sperm was higher for Gpx4^+/U46A^ + Alox15^−/−^ sperm and significant differences were observed when Gpx4^+/U46A^ + Alox15^−/−^ sperm were compared with wild-type (*p* = 0.046) and Gpx4^+/U46A^ (*p* = 0.042) cells. There was, however, no significant difference between wild-type and Gpx4^+/U46A^ cells. Taken together, these data indicate that the combination of functional Gpx4- and Alox15-deficiency induced spontaneous acrosomal exocytosis in capacitated sperm.

When the acrosome reaction was induced by progesterone (Figure 6C, Pg lane), we found that as expected an increased share of wild-type sperm underwent acrosomal exocytosis. In contrast, Gpx4^+/U46A^ sperm were resistant to progesterone treatment. For Gpx4^+/U46A^ + Alox15^−/−^ sperm, progesterone did not induce a further increase in the relative share of post-acrosomal cells. In other words, the degree of spontaneous acrosomal exocytosis of Gpx4^+/U46A^ + Alox15^−/−^ sperm could not be further increased by progesterone. Finally, we treated capacitated sperm of all genotypes with calcium ionophore and observed a strong increase in the relative share of post-acrosomal cells. In fact, under these experimental conditions, the relative share of post-acrosomal sperm was maximal for each genotype. Even the relatively high share of post-acrosomal cells among Gpx4^+/U46A^ + Alox15^−/−^ sperm was increased by calcium ionophore treatment (*p* = 0.064) although the difference showed only borderline significance.

Taken together, these data indicate that Alox15 deficiency on the *Gpx4^+/U46A^* background induces a spontaneous acrosome reaction during the capacitation period (Figure 6A) however for in vitro fertilization the presence and absence of Alox15 in the sperm do not play a major role (Figure 5C). In contrast, the expression of Gpx4 is essential.

### 2.8. Systemic Inactivation of the Alox15 Gene Normalizes the Increased Degree of Total Protein Tyrosine Phosphorylation That Was Elevated by the Expression of the Catalytically Inactive Gpx4^+/U46A^ Mutant

As shown in Figure 5D, G Gpx4^+/U46A^ sperm displayed an elevated degree of total protein tyrosine phosphorylation after capacitation when these cells were compared with the Gpx4^+/+^ wild-type spermatozoa. In contrast, we did not find significant tyrosine phosphorylation differences when wild-type sperm (Gpx4^+/+^) were compared with Alox15 knock-out mice (Figure 6D–F, lane Gpx4^+/+^ + Alox15^−/−)^. However, when the Alox15 knock-out was crossed into the Gpx4^+/U46A^ background (Gpx4^+/U46A^ + Alox15^−/−^), we observed normalization of the total protein tyrosine phosphorylation levels after capacitation compared with Gpx4^+/+^ wild-type or Gpx4^+/U46A^ spermatozoa (Figure 6D–F).

## 3. Discussion

*Gpx4 is modulating the acrosomal exocytosis of sperm*—Previous studies have convincingly shown that Gpx4 is expressed in different subcellular compartments (nucleus, acrosome, mitochondria, and residual bodies) of rat spermatozoa [54,55]. These findings we also confirmed by immunoelectron microscopy in mouse sperm cells (Figure S7). The protein is localized in the midpiece of sperm and contributes to the formation of the mitochondrial capsule [24,25,27]. Together with other sperm proteins, Gpx4 undergoes oxidative polymerization and these protein polymers arrest the mitochondria in the midpiece of the sperm. The function of Gpx4 as a structural protein in spermatozoa was extended by identifying it as an insoluble component of the nuclear and acrosomal matrix [45]. Here we detected Gpx4 in the midpiece but we also observed robust immunohistochemical Gpx4 signals in the anterior region of the head of non-permeabilized resting sperm (Figure 2B). Interestingly, during the capacitation period, which prepares the sperm for the acrosome reaction, we observed an almost complete shift of the Gpx4 staining from the acrosome to the post-acrosomal segment of the sperm (Figure 2B,D). When the acrosome reaction was subsequently induced by progesterone or calcium ionophore, the subcellular distribution pattern of Gpx4 was not altered any further. In other words, sperm capacitation induces major alterations in the subcellular localization of Gpx4 but the acrosome reaction does not. This finding is in agreement with proteomic analyses of boar spermatozoa, where Gpx4 was identified as a protein that plays a role in the capacitation process [56]. In human spermatozoa, Gpx4 was identified as a protein capable of interacting with zona pellucida glycoproteins [57]. This suggests that the multi-functionality of this enzyme is not only related to its function as a structural protein or in detoxification, but also to a role in sperm-egg interaction [58].

The heterozygous expression of catalytically inactive Gpx4 attenuated the intensity of the acrosome reaction (Figure 5B) and reduced the success rate of in vitro fertilization (Figure 5C). This finding suggests a key role of Gpx4 in promoting oocyte fertilization. The molecular mechanism has not been explored in detail but considering the dual character of Gpx4 [25,59], there may be two different mechanistic scenarios.

***First scenario***—Gpx4 functions as a structural protein: as for the formation of the mitochondrial capsule, Gpx4 might be required as a structural protein for the acrosome reaction. The formation of the mitochondrial capsule involves oxidative protein polymerization [24,25,60] and as a consequence of this reaction, an immobilizing mixed polymeric protein structure is formed, which arrests the mitochondria in the midpiece. We observed by immunohistochemical staining that in resting sperm the Gpx4 is present in the anterior region of the head which harbors the acrosome but shifts to the post-acrosomal segment during the capacitation reaction. It appears unlikely that large protein polymers can easily move inside cells. In fact, midpiece staining remained unaltered during capacitation and acrosomal exocytosis and these data suggest a high degree of immobility of the capsule proteins.

***Second scenario***—Gpx4 is an anti-oxidative enzyme capable of reducing complex hydroperoxy lipids [32,33]. The crystal structure of this selenoenzyme has recently been solved and a bicyclic reaction mechanism was suggested [61]. Gpx4 reduces the oxidative potential of cells and/or subcellular compartments, and thus initiates or terminates a number of physiological processes including the activation and inactivation of redox-sensitive transcription factors [62,63]. Sperm are sensitive to oxidative stress because of their high PUFA membrane content [64] and balanced peroxidation of membrane lipids is essential for sperm motility [65] as well as for capacitation [53] and the acrosome reaction [66]. Sperm lysates prepared from Gpx4^+/U46A^ animals only exhibited about 65% of Gpx4 activity when compared with sperm lysates prepared from wild-type sperm (Figure S9A) and this data suggests that Gpx4^+/U46A^ sperm have a compromised anti-oxidative defense system favoring oxidative stress. Oxidative stress plays an important role in the development of spermatozoa [67] and is necessary to induce sperm capacitation. Only the generation of controlled amounts of reactive oxygen species supports correct sperm function [68]. Tyrosine phosphorylation is an essential step in sperm capacitation [56,69,70], and the degree of tyrosine phosphorylation is increased when reactive oxygen species are formed [52,53]. Our finding that the heterozygous expression of catalytically inactive Gpx4 (Gpx4^+/U46A^) in spermatozoa augmented the degree of tyrosine phosphorylation during in vitro capacitation (Figure 5D,E) is consistent with these findings. On the other hand, we observed that the heterozygous expression of the catalytically inactive Sec46Ala Gpx4 mutant impairs acrosomal exocytosis (Figure 5A,B). Although spermatozoa from Gpx4^+/U46A^ mice exhibit enhanced tyrosine phosphorylation, the in vitro-induced acrosome response of capacitated spermatozoa is impaired. Reduced Gpx4 activity in spermatozoa may lead to an imbalance in the antioxidant protection system and oxidative changes in membrane lipids and proteins would be the consequence [22,52,53].

In addition to the activity assays, we performed Western blot analyses using lysates of wild-type sperm prepared under native and denaturing conditions. Here we detected a clear immunoreactive band in the 18 kDa molecular weight region suggesting the presence of monomeric Gpx4 in the lysate of native swim-out sperm. Such an immunoreactive band was also observed when the lysate of swim-out sperm was prepared under denaturing conditions. Similar bands were also detected in the lysates of non-capacitated and capacitated sperm when the lysates were prepared under denaturing conditions (Figure S9B).

*Expression of Alox15 in sperm*—The expression of Alox15 in mammalian cells is highly regulated. Immunohistochemical staining indicated major constitutive expression in immature red blood cells, broncho-epithelial cells, and eosinophils [71,72]. In circulating monocytes, the enzyme is not expressed but the in vitro maturation of these cells in the presence of interleukins -4 and -13 induced the dramatic upregulation of Alox15 expression [73,74]. Young rabbit reticulocytes are devoid of functional ALOX15 but the corresponding mRNA is present as a functionally silent RNA-protein complex [75]. During the in vitro maturation of young reticulocytes, the regulatory proteins are degraded and the ALOX15 mRNA is translated into the catalytically active protein [76]. After the enzyme has fulfilled its biological function, it undergoes self-inactivation and is subsequently proteolytically degraded. Thus, mature erythrocytes do not contain ALOX15 anymore [77,78]. Taken together, these data indicate that ALOX15 can only be detected in erythroid cells during a rather narrow time window. In sperm, a similar situation might exist. Immunohistochemical studies indicated that Alox15 is localized in the cytoplasmic droplet [20,21], a specialized organelle that is temporarily formed during the late stages of sperm development. Moreover, ALOX15 expression was shown within the anterior region of the head and in the flagellum of human sperm [79]. Unfortunately, for these staining experiments, control staining with Alox15-deficient sperm has not been performed, and thus, the specificity of the staining signals has not been tested. When we carried out similar staining in Alox15^+/+^ and Alox15-deficient (Alox15^−/−^) sperm, we did not observe specific staining patterns. Although our immunohistochemical studies did not provide clear-cut evidence for Alox15 expression in mouse sperm, analysis of the reaction products Figure 4 and Figure S5 suggested the catalytic activity of the enzyme during sperm development. On the other hand, these data do not necessarily mean that Alox15 is present in mature cauda sperm. It might well be that the enzyme is expressed in earlier developmental stages and that the products survived the developmental process as a signature for previous Alox15 activity.

**Scheme 1:**
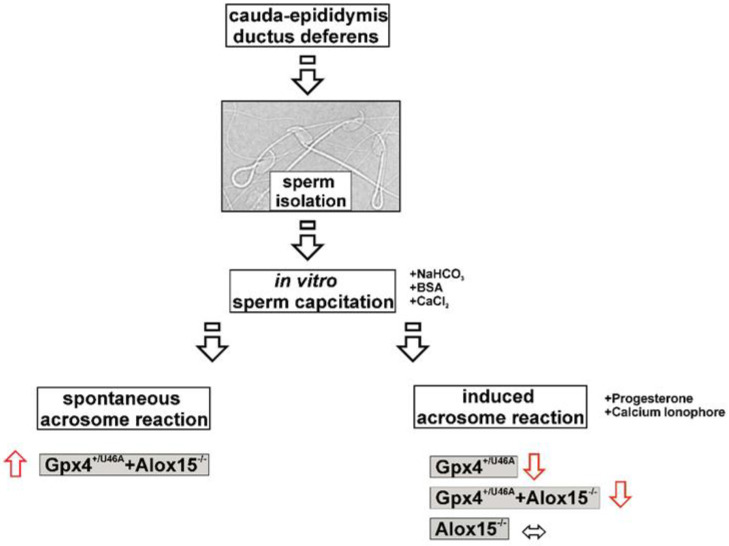
The capacitation process is essential for preparing sperm for acrosomal exocytosis. Sperm isolated from the ductus deferens or cauda epididymis are not susceptible to the induction of the acrosome reaction. However, incubation with sodium bicarbonate, bovine serum albumin, and calcium chloride renders the cells susceptible to induction of the acrosome reaction, which is most frequently initiated by progesterone or calcium ionophore in vitro. The heterozygous expression of a *Gpx4^+/U46A^* allele attenuated the degree of acrosome reaction and a similar phenotype was observed for *Gpx4^+/U46A^* + *Alox15^−/−^* sperm. Interestingly, *Gpx4^+/U46A^* + *Alox15^−/−^* sperm undergo premature acrosomal exocytosis during the in vitro capacitation reaction, which may contribute to the attenuated susceptibility for the normal acrosome reaction.

*Interplay of Gpx4 and Alox15 during capacitation and acrosome reaction*—The successful fertilization of oocytes in vitro and in vivo is a complex process that involves a number of different steps. Sperm prepared from the epididymal cauda or the ductus deferens are not ready for induction of the acrosome reaction. As shown in Scheme 1, they must first undergo capacitation to prepare themselves for induction of the acrosome reaction.

Capacitated sperm can then be treated with progesterone or calcium ionophore to induce acrosomal exocytosis, which is essential for oocyte fertilization. Systemic heterozygous expression of a catalytically silent Gpx4 impaired the intensity of the acrosome reaction (Figure 5B) and makes the sperm less suitable for in vitro fertilization (Figure 5C). The acrosome reaction of Gpx4-deficient sperm induced by calcium ionophore was less severely inhibited by the genetic manipulation when compared with the experiments, in which progesterone was used as an inducer of the acrosome reaction. Under progesterone treatment, Gpx4 deficiency reduced the degree of acrosomal exocytosis by about 75%. When we carried out similar calculations for calcium ionophore-induced acrosomal exocytosis, Gpx4 deficiency reduced the degree of acrosomal exocytosis by about 47%. The molecular reasons for this difference have not been explored in this study and should be addressed in follow-up experiments. Acrosomal exocytosis is induced by the elevation of cytosolic calcium concentration [2]. As an artificial inducer of the acrosome reaction, calcium ionophore directly increases the cytosolic calcium concentrations by carrying calcium ions across the cellular membranes. In contrast, as a physiological inducer, progesterone elevates the cytosolic calcium concentrations indirectly via the receptor-mediated activation of ion channels [80]. Our observation that Gpx4 deficiency induced a more pronounced difference when acrosomal exocytosis is induced by progesterone suggested that the calcium entry into the sperm may be affected by our genetic manipulation. However, it remains unclear for the moment which ion channels may be involved and how Gpx4 deficiency may impact these channels.

The involvement of Alox15 in normal sperm functionality has been demonstrated by pharmacological interference studies [37,79] and by supplementation studies of sperm with specific lipoxygenase products [81]. Whereas the inhibition of lipoxygenase activity inhibits the acrosome reaction, the incubation of cells with 15-HETE stimulated acrosomal exocytosis. In contrast, systemic Alox15 deficiency on a wild-type background (Alox15^−/−^ sperm) hardly impacted the acrosome reaction (Figure S4). However, when Alox15 deficiency was crossed into *Gpx4^+/U46A^* mice, the sperm of these animals (Gpx4^+/U46A^ + Alox15^−/−^) underwent spontaneous acrosome reaction during the capacitation period (Figure 6C). Interestingly, the addition of progesterone did not lead to a further increase in acrosomal exocytosis. The impaired acrosome reaction in Gpx4^+/U46A^ sperm could not be prevented by an additional Alox15 knockout in vitro. However, since the systemic inactivation of the Alox15 gene normalized the elevated level of the total protein tyrosine phosphorylation that was increased by an expression of the catalytically inactive Gpx4^+/U46A^ mutant (Figure 6D,E), an involvement of Alox15 in the redox homeostasis in sperm could be suggested.

Translating these findings to the in vivo situation, it might be hypothesized that Gpx4^+/U46A^ + Alox15^−/−^ sperm may undergo premature acrosomal exocytosis before they get in contact with an oocyte and impair male fertility. This hypothesis is consistent with the results of our in vitro fertilization experiments. When Alox15-deficient oocytes (wild-type in the Gpx4 locus) and Alox15-deficient sperm (also wild-type in the Gpx4 locus) were used, we observed normal rates of in vitro fertilization (Figure 6A). In contrast, when the same oocytes were incubated with sperm, which carries the U46A-mutant *Gpx4* allele in addition to the *Alox15* knockout allele, in vitro fertilization was prevented. These data indicate that functional Gpx4 deficiency combined with lacking Alox15 expression strongly impairs sperm functionality under in vitro conditions. This impairment may mainly be related to the spontaneous acrosome reaction, which leads to the loss of acrosomes before the sperm reach the oocyte. It should be noted that in vitro studies cannot fully mirror the in vivo processes. It was shown that mouse spermatozoa have already completed the acrosome reaction when it reaches the oocyte [82,83]. This is in line with our previous in vivo research, where we have shown that male fertility caused by the heterozygous expression of catalytically inactive Gpx4 can be rescued by the systemic inactivation of Alox15 [27].

In this study, we show that Gpx4 deficiency not only impairs sperm motility but also impacts capacitation-related protein tyrosine phosphorylation and reduces the extent of the acrosome reaction. Thus, the molecular reasons for the infertility of Gpx4-deficient individuals may not only be related to motility problems of the sperm but also to their inability to induce proper acrosome reaction for fertilization. If one translates these findings into an in vivo situation, one can conclude that even when a Gpx4-deficient sperm reaches an oocyte it cannot penetrate the zona pellucida of the oocyte-cumulus complex, and thus, oocyte fertilization is not possible. Additional systemic inactivation of the Alox15 gene in Gpx4-deficient sperm normalizes protein tyrosine phosphorylation, but this is not sufficient to rescue the acrosome reaction. Instead, this additional modification leads to a spontaneous acrosome reaction during the capacitation process.

## 4. Materials and Methods

*Chemicals*—The chemicals were from the following sources: HPLC standards of different polyenoic fatty acids were purchased from Cayman Chem. (distributed by Biomol GmbH, Hamburg, Germany). HPLC solvents from Fisher Scientific (Schwerte, Germany), oligonucleotide synthesis from BioTez (Berlin, Germany), progesterone and calcium ionophore A23187 from Sigma (Saint Louis, MI, USA).

*Animals and breeding*—All the mice were bred and maintained in a specific pathogen-free (SPF) animal facility on a 14h/10h LD cycle with food and water ad libitum. Procedures were performed according to the EU Directive 2010/63/EU, Federation of Laboratory Animal Science Associations, and the local guidelines. All animal experiments were performed in compliance with the German animal welfare law and have been approved by the institutional committee on animal experimentation (VF experiments: A 0025/17; Organ/Sperm analysis: T 0437/08, T-CH 0003_22). *Alox15* knockout mice [39] were obtained from Jackson Laboratory (Bar Harbor, ME, USA) and backcrossed for 8 generations into C57BL/6 background. Gpx4^+/U46A^ (Gene ID: 625249) mice were created as described before [48] and corresponding wild-type control animals were outbred by mating heterozygous allele carriers. Separate colonies of Gpx4^+/U46A^ mice and outbred wild-type controls were maintained. Alox15 (Gene ID: 11687) deficiency was bred into Gpx4^+/U46A^ mice by repeated crossing of these animals with Alox15^−/−^ mice and subsequent intercrossing heterozygous allele carriers. All individuals were genotyped for the two gene loci and Gpx4^+/U46A^ + Alox15^−/−^ mice were used.

*PCR genotyping*—Genomic DNA was prepared from ear punch using Invisorb Spin Tissue Mini Kit (Invisorb, Berlin, Germany) and genomic PCR was carried out with MyTaq™Red Mix (BIOLINE, Heidelberg, Germany) system. Primers used for analyzing the Gpx4 gene locus were as follows: up-5′-GACAGATGGCTCTGGACCTGGGTG-3′, do-: 5′-TAATCTGGCGTGGTAGGGGCA GAC-3′. The wild-type allele showed up as a 412 bp fragment but the *Sec46Ala* mutant allele gave an amplification product of higher molecular weight (587 bp). The higher molecular weight was due to a residual LoxP/FRT sequence in the genome after deletion of the neo-cassette. Heterozygous allele carriers showed two bands in PCR. For genotyping of the *Alox15* locus, we employed the primer combination recommended by Jackson laboratory: oIMR0013 Neo: 5′-CTTGGGTGGAGAGGCTATTC-3′, oIMR0014 Neo: 5′-AGGTGAGATGACAGGAGATC-3′, oIMR0862-Exon3: 5′-CGTGGTTGAAGACTCTCAAGG-3′, oIMR0863-Exon4: 5′-CGAAATCGCTGGTCTACA GG-3′. The wild-type band was 229 bp and the knockout band was 280 bp. Homozygous allele carriers only showed the band with 280 bp.

*Preparation of sperm*—For preparation of non-capacitated sperm, 10–16 week old mice were sacrificed by cervical dislocation after isoflurane anesthesia. Epididymal cauda and vas deferens were excised and surrounding tissue and blood vessels were removed. The tissue was transferred to a petri dish containing the following buffer in the absence of calcium, BSA, and sodium bicarbonate: 25 mM HEPES, 109 mM NaCl, 4.77 mM KCl, 1.19 mM MgSO_4_, 5.56 mM glucose, 25.94 mM sodium lactate, 1.19 mM KH_2_PO_4_, 1 mM sodium pyruvate, pH 7.4, and then cut into pieces and allowing the sperm to disperse for 10 min at 37 °C and the suspension was filtered through a 30-µm nylon mesh (Partec, Goerlitz, Germany). This medium does not support capacitation (NC-medium). The sperm vitality was tested with Eosin B (0.05%) staining and 100 sperm cells were evaluated. After swimming out, spermatozoa concentration was assessed with a Neubauer chamber.

*Sperm capacitation and acrosome reactionanalysis*—Non-capacitated caudal epididymal sperm were washed in 2 mL of NC-medium by centrifugation at 800× *g* for 10 min at room temperature. The sperm were resuspended to a final concentration of 5–10 × 10^6^ cells/mL in fresh NC-medium. For sperm cell capacitation, a sperm aliquot of the same preparation was resuspended in NC-medium supplemented with BSA (3 mg/mL), NaHCO_3_ (25 mM), CaCl_2_ (1.17 mM), and incubated for 45–60 min at 37 °C in 95% humidified air and 5% CO_2_. To induce the acrosome reaction, progesterone (50 µM) or calcium ionophore A23187 (20 µM) was added to approximately 5 million capacitated and non-capacitated sperm cells (negative control). Spermatozoa were then incubated for 40 min at 37 °C. Negative solvent control was incubated with DMSO under the same conditions. Sperm cells were centrifuged at 500× *g* for 10 min at room temperature, washed with Dulbecco’s phosphate-buffered saline (DPBS), and fixed with 5% paraformaldehyde for 10 min at room temperature. The fixed sperm were washed with DPBS and 10 µL aliquots were air dried onto slides. Staining was conducted with Coomassie solution (0.22% Coomassie Blue in 50% methanol and 10% acetic acid) for 2 min. The slides were then gently washed and mounted with 50% glycerol. At least 200–300 stained cells/treatment were scored using Axioskop microscope (Zeiss, Jena, Germany) with a 40-fold objective clear field. Heads of sperm with the acrosome were stained dark blue (intact, not reacted acrosome) and without acrosome (no stained, reacted acrosome). The percentage of capacitated and non-capacitated acrosome-reacted spermatozoa was calculated.

*Protein extraction and immunodetection of phosphotyrosine residues*—Freshly prepared spermatozoa (swim-out) or spermatozoa incubated in the respective medium (non-capacitating-NC, capacitating-Cap) were washed with 1 mL PBS and 2 × 10^6^ sperm were resuspended in 2× ProSieve^TM^ ProTrack™Protein Loading Buffer (Lonza, Basel, Switzerland) without reducing agent. Samples were boiled for 5 min and centrifuged for 3 min at 5000× *g*. The supernatant was recovered and boiled again in the presence of 5% ß-mercaptoethanol. Solubilized proteins (corresponding to 1 × 10^6^ spermatozoa/lane) were analyzed by SDS-PAGE on a 7.5% polyacrylamide gel. Separated proteins were transferred to a 0.45 µm nitrocellulose membrane (Thermo Scientific GmbH, Schwerte, Germany) by a wet blotting method (ProSieve Ex Western Blot Transfer Buffer, dilution of a 10-fold concentrated stock solution, Biozym Scientific GmbH, Hessisch-Oldendorf, Germany). The membranes were blocked with blocking solution (dilution of a 10-fold concentrated stock solution, BlueBlock PF for Blotting, SERVA Electrophoresis GmbH, Heidelberg, Germany), washed three times with PBS/0.3% TWEEN 20, and incubated with an anti-phosphotyrosine-HRP antibody (pY20, ThermoFischer Scientific, Dreieich, Germany) for 1–2 h at room temperature. After several steps of washing, the membranes were stained using the SERVALight Polaris CL HRP WB Substrate Kit (SERVA Electrophoresis GmbH, Heidelberg, Germany) for 5 min at room temperature. Chemiluminescence was quantified using the FUJIFILM Luminescent Image Analyzer LAS-1000plus (Fujifilm Europe GmbH, Düsseldorf, Germany). As loading control, membranes were developed with anti-alpha tubulin antibody (DM1A, ThermoFischer Scientific, Dreieich, Germany). For quantification, the intensities of the total protein tyrosine phosphorylation levels under the appropriate conditions were quantified using ImageJ and normalized to the respective intensity of alpha tubulin.

*Immunocytochemistry*—To explore subcellular distribution of Gpx4 we employed an anti-GPX4 antibody that was raised against the pure recombinant U46A GPX4 mutant. The antibody recognizes a specific sequence epitope [84], which is conserved between human and mouse GPX4, and thus, the antibody cross-reacts with mouse Gpx4. The specificity was shown by immunoblotting of Gpx4-transfected HEK293 cells and corresponding mock-transfectants (Figure S8). This antibody is commercially available from Merck, MABF1969 (Darmstadt, Germany). For immunohistochemical staining cells were fixed using 4% paraformaldehyde. After extensive washing cells were put onto poly-L-ornithine glass slides, which were subsequently blocked with 3% bovine serum albumin (BSA) containing PBS. Then primary antibody was added (5 μg/mL) and the samples were incubated overnight at 4 °C [84]. A fluorescence labeled goat anti-mouse IgG secondary antibody 1:100 (Alexa 594, ThermoFisher, Schwerte, Germany) was applied for 1 h at room temperature. All glass slides were mounted with Fluoromount onto coverslips. Gpx4 labeled spermatozoa were then examined using an EVOS Cell Imaging Systems (EVOS M5000, ThermoFisher Scientific, Darmstadt, Germany) with a 100-fold immersion objective. At least 80–100 stained cells per treatment were examined for acrosomal scoring or post acrosomal localization of Gpx4-IHC signals and the percentages of the score and localization were calculated.

To detect Alox15, we employed two different antibodies: (i) the homemade ALOX15 antibody that was raised against the native rabbit ALOX15 which has previously been shown to cross-react with mouse and human Alox15. (ii) A commercial anti-12-lipoxygenase (mouse leukocyte-type 12-lipoxygenase) antibody (Cat No. 160304, Cayman, MI, USA). This antibody was raised against a peptide of mouse leukocyte-type 12-lipoxygenase (mouse Alox15). In Western blotting of peritoneal macrophage lysates, both antibodies showed an immunoreactive protein band in the 70 kDa molecular weight range suggesting that both antibodies recognize mouse Alox15. When lysates of peritoneal macrophages of Alox15^−/−^ mice were analyzed as controls we did not see an immunoreactive band with our homemade ALOX15 antibody but detected a pronounced band with the commercial antibody. These data indicate that this commercial anti-Alox15 antibody may also pick up non-Alox15 proteins in the appropriate molecular weight region, and thus, we decided to employ our homemade anti-Alox15 antibody for this study.

*Gpx4 Activity Assay*—Freshly prepared sperm cells from Gpx4^+/+^ (wild-type) and Gpx4 ^+/U46A^ animals were lysed in 0.4 mL of lysis buffer (50 mM Tris-HCl, 0.3 mM KCl, 1 mM EDTA, 10% glycerol, 5 mM TCEP, 1% Triton X100, protease inhibitory mix (SERVA, Heidelberg, Germany) pH 7.5). After centrifugation at 10,000× *g* for 10 min, the supernatants were employed for Gpx4 activity assays. Gpx4 activity was determined spectrophotometrically employing the coupled optical test [48]. The assay mixture was composed of 1 mL 0.1 M Tris/Cl buffer, pH 7.4, containing 5 mM ethylenediaminetetraacetic acid, 0.1% Triton X-100, 0.2 mM NADPH, 3 mM glutathione, and 1 U glutathione reductase. Aliquots of 10,000 g lysate supernatants of sperm cell homogenates (Gpx4^+/+^ or Gpx4^+/U46A^ mice) were added, and the assay mixture (absence of substrate) was preincubated at 37 °C for 5 min. Then, the reaction was initiated by the addition of 25 µM phosphatidylcholine hydroperoxide, and the decrease in absorbance at 340 nm was recorded (molar extinction coefficient for NADPH of 6.22 × 10^6^ (M × cm)^−1^).

*Immunoblotting*—Aliquots of total sperm protein extracts (see above “*Protein extraction and immunodetection of phosphotyrosine residues*”) of wild-type spermatozoa (swim-out, non-capacitated, capacitated) and sperm protein lysate prepared for Gpx4 activity assay were analyzed by SDS-PAGE on a 7.5% polyacrylamide gel. Separated proteins were transferred to a 0.45 µm nitrocellulose membrane (Thermo Scientific GmbH, Schwerte, Germany) by a wet blotting method (ProSieve Ex Western Blot Transfer Buffer, dilution of a 10-fold concentrated stock solution, Biozym Scientific GmbH, Hessisch-Oldendorf, Germany). The membranes were blocked with blocking solution (dilution of a 10-fold concentrated stock solution, BlueBlock PF for Blotting, SERVA Electrophoresis GmbH, Heidelberg, Germany) and washed three times with PBS/0.3% TWEEN 20 and incubated overnight with the monoclonal anti-human Gpx4 antibody (#MABF1969, Merck, Darmstadt, Germany) [84]. The membrane was washed with PBS/0.3% Tween 20 and incubated with secondary antibodies (anti-mouse: Sigma or anti-rabbit: Santa Cruz, Heidelberg, Germany) for 30 min. After several steps of washing, the membranes were stained using the SERVALight Polaris CL HRP WB Substrate Kit (SERVA Electrophoresis GmbH, Heidelberg, Germany) for 5 min at room temperature. Chemiluminescence was quantified using the FUJIFILM Luminescent Image Analyzer LAS-1000plus (Fujifilm Europe GmbH, Düsseldorf, Germany).

*RNA extraction and qRT-PCR of Alox mRNA*—Total RNA was extracted from the testis, epididymis, and sperm from cauda and vas deferens using the NucleoSpin RNA Plus (MACHEREY-NAGEL, Düren, Germany). Synthesis of cDNAs was performed with 0.1–2 µg of the total RNA preparations using oligo(dT) primers and Tetro Reverse Transcriptase (BIOLINE, Luckenwalde, Germany). Quantitative real-time PCR (RT-PCR) was carried out with a Rotor-Gene 3000 (Corbett Research) using the SensiFast SYBR PCR Kit (BIOLINE, Luckenwalde, Germany). The following primer sets were employed to quantify the Gapdh, Alox15, Alox12 (Gene ID: 11684), Alox5 (Gene ID: 11689, Gapdh; 5′-CCATCACCATCTTCCAGGAGCGA-3′; 5′-GGATGACCTTGCCCACAGC-CTTG-3′, Alox15; 5′-GCTGCACCGTGGTTGAAGACTCT-3′; 5′-CTGTACAGACTCCTCCTTTCTTCC-3′, Alox12; 5′-GCGGCCATGTTCAGTTGCTTAC-3′; 5′-CATCGTCACGTCGTCCTTGCTG-3′. The Rotor-Gene Q software was used to analyze the PCR raw data. Specific amplicons were prepared as external amplification standards for all target (Gpx4 and Alox isoforms) and reference (Gapdh) genes and this procedure allowed exact quantification of the mRNA copy numbers. Gapdh mRNA was used as an internal amplification standard and expressions of the target genes were normalized to the Gapdh content. Analyses were performed in triplicate and mean ± standard deviations are given.

*Determination of the PUFA composition of sperm lipids*—Sperm were prepared as described above and total sperm lipids were extracted with the Bligh–Dyer method [85]. The solvent was evaporated using a rotatory evaporator, lipids were reconstituted in 0.35 mL methanol and 0.075 mL of anaerobic 40% KOH was added. Afterward, the ester lipids were then hydrolyzed (20 min at 60 °C) and the hydrolysis mixture was neutralized with concentrated acetic acid (0.075 mL). Precipitate was spun down and aliquots were injected to RP-HPLC, which was carried out on a Shimadzu LC-20 instrument equipped with a SIL-20AC autoinjector and an SPD-M20A diode array detector. For PUFA analysis, we recorded the absorbance at 210 nm. A Nucleodur C18 Gravity column (Macherey-Nagel, Düren, Germany; 250 × 4 mm, 5 µm particle size) was used and fatty acid derivatives were eluted with a solvent system consisting of acetonitrile/water/acetic acid (70/30/0.1, by vol.) at a flow rate of 1 mL/min. The retention times of different PUFA standards (Figure S1) were determined and chromatographic scale was calibrated by injecting known amounts of these standards.

*Quantification of oxygenated fatty acids in the hydrolyzed lipid extracts of sperm*—LC/MS/MS analyses of targeted oxylipins were performed on a Shimadzu Nexera liquid chromatography system coupled with a 6500 QTrap mass spectrometer (Sciex) [86,87]. Briefly, liquid chromatography was carried out at 45 °C on a Zorbax Eclipse Plus C18 column (Agilent Technologies, 150 × 2.1 mm, 1.8 μm) at a flow rate of 0.5 mL/min over a time period of 22.5 min. Mobile phase A was a water/mobile phase B mixture (95/5; *v*/*v* plus 0.1% acetic acid) and mobile phase B was an acetonitrile/methanol mixture (800/150; *v*/*v* containing 0.1% acetic acid). For analysis, a linear gradient of mobile phase B was used: 30% for 1 min, 30–35% from 1 to 4 min, 35–67.5% from 4 to 12.5 min, and 67.5–100% from 12.5 to 17.5 min. Then the gradient was held at 100% mobile phase B for 3.5 min. Finally, the column was re-equilibrated to initial conditions. Injection volume was 5 µL. Ionization was performed using electrospray ionization in the negative mode (ESI-) with the following parameters: temperature 475 °C, N2 as drying gas, ion source gas1 60 psi, ion source gas2 60 psi, curtain gas 35 psi, ESI spray voltage −4.5 kV. Cycle time was 0.4 s. Quantification was performed using external calibration curves with authentic standards for all measured oxylipins and deuterated internal standards. The list of analyzed oxylipins as well as limit of detection, limit of quantification, retention time, and calibration range is provided in Table 1 below.

*In vitro fertilization*—In vitro fertilization of mouse oocytes was carried out as described in [88]. In brief, cumulus-oocyte complexes (COCs) were isolated from superovulated female mice, which received an intraperitoneal injection of 5 IU pregnant mare serum gonadotropin (5 IU at 2000; Intergonan, Intervet, Unterschleißheim, Germany) and 46 h later an i.p. injection of 5 IU of human chorionic gonadotropin (Ovogest, Intervet). 12 to 14 h later the mice were sacrificed by cervical dislocation, the oviducts were prepared, the cumulus-oocyte complexes were removed from the ampullae and coincubated in IVF medium (K-RVFE-50, Cook Medical Europe, Baesweiler, Germany) with approximately 3.7 × 10^6^ capacitated fresh epididymal spermatozoa for 4 to 5 h in an incubator (CB 60, Binder, Tuttlingen, Austria). Sperm were extracted from the epididymal cauda as described [88]. After coincubation, the oocytes were washed and cultured (K-RVFE, Cook Medical Europe, Baesweiler, Germany) overnight for 20 h in an incubator. For quantification, morphologically intact 2-cell embryos that had 2 blastomeres of approximately the same size with homogeneous cytoplasm, intact zonae pellucida with no blebbing nor fragmenting were selected by using a stereomicroscope (MZ12.5, Leica, Wetzlar, Germany) under 40× magnification.

*Statistics*—Statistical analyses were carried out with the SPSS 23 (IBM, New York, NY, USA) software package and the results are presented as medians. For experiments, in which the data are not normally distributed, a two-tailed Mann–Whitney U test was employed. Significance was accepted at *p* < 0.05. Within the boxplots, the black vertical lines indicated the median. The 25th and the 75th percentile are visualized as upper and lower box limits. For normally distributed data the Student *t*-test was used.

## 5. Conclusions

The anti-oxidant enzyme Gpx4 is present in the anterior region of the head of resting mouse sperm and translocates to the post-acrosomal segment during sperm capacitation. Together with its functional counterplayer Alox15, it plays regulatory roles during capacitation, acrosomal exocytosis, and oocyte fertilization.

## Figures and Tables

**Table 1:** LC/MS/MS analyses of targeted oxylipins.

Analyte	Internal Standard (IS)	Retention Time (min)	Molecular Mass (g/mol)	Mass Transition		LOD	Calibration Range (nM)	
				Q1 (*m*/*z*)	Q3 (*m*/*z*)	(nM)	Lower	Upper
12-HETE	12(S)-HETE-d8	14.4	320.5	319.2	179.2	0.3	3	2000
14-HDHA	15(S)-HETE-d8	14.2	344.5	343.2	205.2	0.3	1	2000
9-HOTrE	13(S)-HODE-d4	12.4	294.4	293.2	171.2	0.3	1	2000
13-HOTrE	13(S)-HODE-d4	12.6	294.4	293.2	195.1	1	3	2000
14-HPHA	12(S)-HETE-d8	14.9	346.5	345.2	205.2	3	6	6000
13(S)-HODE-d4	-	13.6	300.5	299.5	198.1	-	-	-
12(S)-HETE-d8	-	14.4	328.2	327.2	184.2	-	-	-
15(S)-HETE-d8	-	14.0	328.2	327.2	226.2	-	-	-

## Data Availability

The original experimental data obtained in the study can be obtained free of charge upon request from the corresponding author.

## Supplementary Materials

The supporting information can be downloaded at: https://www.mdpi.com/article/10.3390/ijms23179907/s1.

## Abbreviations

A	alanine
Ala	alanine
Alox15	mouse arachidonate 15-lipoxygenase
AA	5,8,11,14-eicosatetraensäure
DHA	4,7,10,13,16,19-docosahexaenoic acid
DPA	7,10,13,16,19-docosapentaenoic acid
EPA	5,8,11,14,17-eicosapentaenoic acid
Gpx4	glutathione peroxidase 4
H(p)ETE	hydro(pero)xyeicosatetraenoic acids
PUFA	polyenoic fatty acid
Sec	selenocysteine
Ser	serine
U	selenocysteine
