# Supplementary material for: Unbalanced Expression of Glutathione Peroxidase 4 and Arachidonate 15-Lipoxygenase Affects Acrosome Reaction and In Vitro Fertilization
Source: Int J Mol Sci. 2022 Aug 31;23(17):9907. doi: 10.3390/ijms23179907 (PMC9456195; doi:10.3390/ijms23179907)
Supplement: Supplementary file 1 [file ijms-23-09907-s001.zip › ijms-1836017-supplementary.pdf]

Supplementary Materials to the Article

Unbalanced expression of glutathione peroxidase 4 and arachidonate 15-lipoxygenase affects acrosome reaction and in vitro fertilization

Mariana Soria-Tiedemann [1], Geert Michel [2], Iris Urban [2], Maceler Aldrovandi [3,4], Valerie B O'Donnell [3], Sabine Stehling [1], Hartmut Kuhn [1,] and Astrid Borchert [1*]

1 Department of Biochemistry, Charité - Universitätsmedizin Berlin, corporate member of Freie Universität Berlin and Humboldt-Universität zu Berlin, Charitéplatz 1, 10117 Berlin, Germany
2 Department of Transgenic Technologies, Charité - Universitätsmedizin Berlin, corporate member of Freie Universität Berlin and Humboldt-Universität zu Berlin, Lindenberger Weg 80, D-13125 Berlin, Germany
3 Systems Immunity Research Institute, School of Medicine, Cardiff University, Cardiff, CF14 4XN, UK
4 Helmholtz Zentrum München, Institute of Metabolism and Cell Death, Ingolstädter Landstr. 1, Neuherberg, 85764, Germany
* Correspondence: astrid.borchert@charite.de (A.B.); Tel.: +49-30-450-528-034 (A.B.)

1. Supplemental Figure S1: HPLC analysis of polyunsaturated fatty acid standards

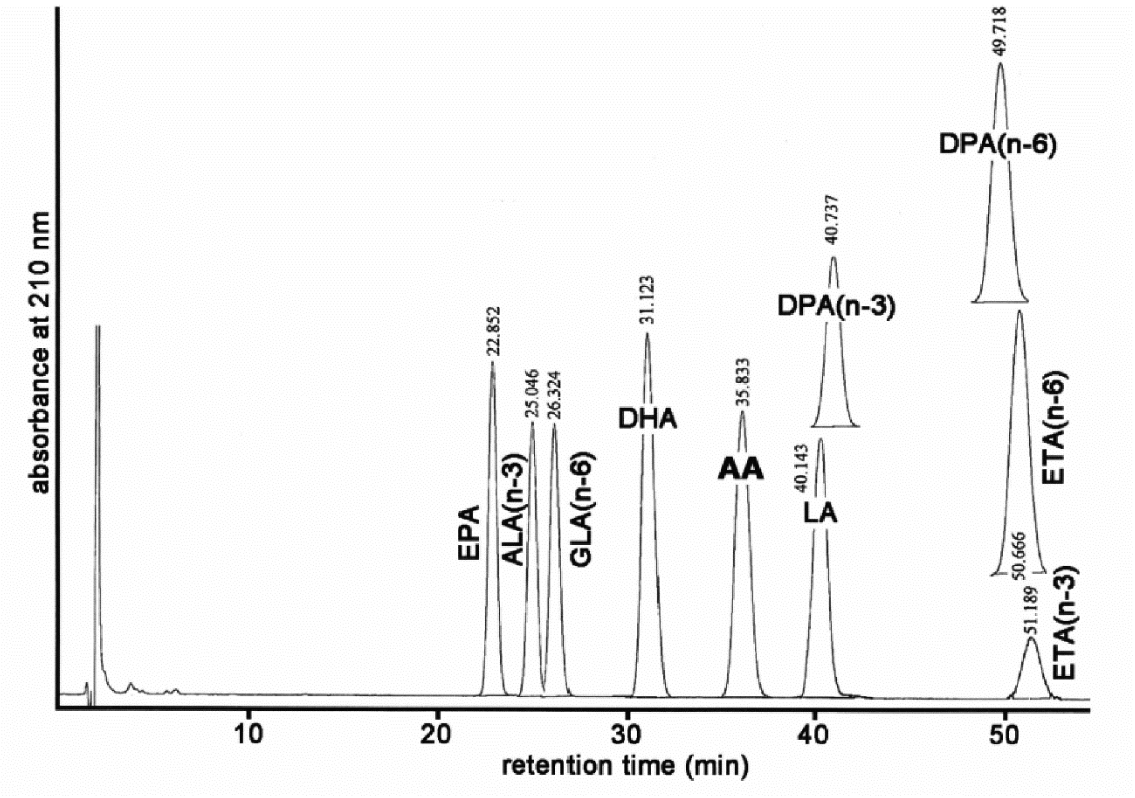

Figure S1. Retention times of different PUFA standards. HPLC was performed on a Shimadzu LC-20 instrument equipped with a SIL-20AC autoinjector and a SPD-M20A diode array detector. For PUFA analysis we recorded the absorbance at 210 nm. A Nucleodur C18 Gravity column (Macherey-Nagel, Düren, Germany; 250 x 4 mm, 5 µm particle size) was used and fatty acid derivatives were eluted with a solvent system consisting of acetonitrile/water/acetic acid (70/30/0.1, by vol.) at a flow rate of 1 ml/min. The retention times of different PUFA standards were determined and chromatographic scale was calibrated by injecting known amounts of these standards.

2. Supplemental Figure S2: HPLC analysis of polyunsaturated fatty acids in sperm

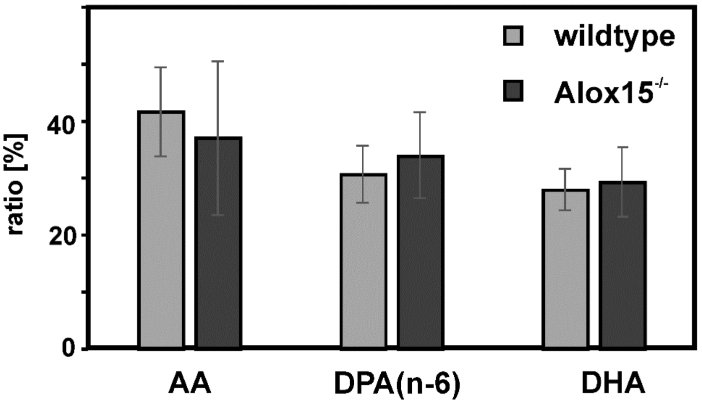

Figure S2. Quantification of main PUFAs in sperm lipids. Sperm lipids were extracted and ester lipids were hydrolyzed as described in Materials and Methods. HPLC was performed on a Shimadzu LC-20 instrument equipped with a SIL-20AC autoinjector and a SPD-M20A diode array detector. For PUFA analysis we recorded the absorbance at 210 nm. A Nucleodur C18 Gravity column (Macherey-Nagel, Düren, Germany; 250 x 4 mm, 5 μm particle size) was used and fatty acid derivatives were eluted with a solvent system consisting of acetonitrile/water/acetic acid (70/30/0.1, by vol.) at a flow rate of 1 ml/min. The retention times of AA (arachidonic acid), DPA (docosapentaenoic acid), and DHA (docosahexaenoic acid) standards were determined and chromatographic scale was calibrated by injecting known amounts of these standards (**Figure S1**) and the ratios were estimated. Significances were calculated using Student`s *t*-test. N=12 animals from each genotype (wildtype-C57BL/6J and Alox15$^{-/-}$) were analyzed and *p*-values <0.05 were considered statistically significant.

3. Supplemental Figure S3. Expression of selected mouse Alox isoforms in different organs of the male reproductive system.

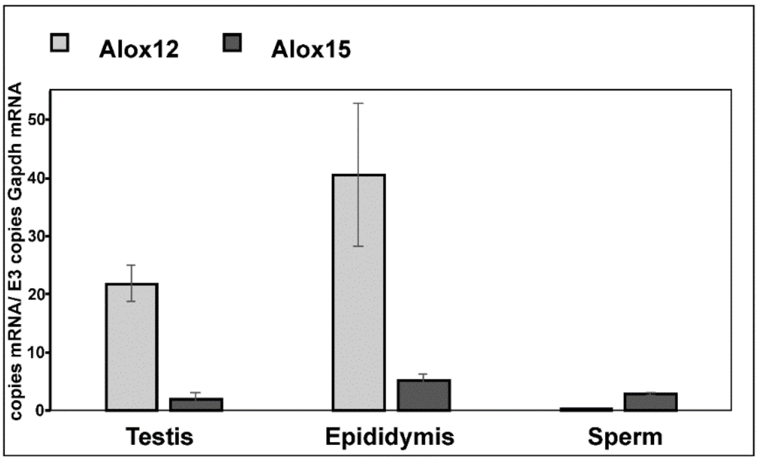

Figure S3. Expression of Alox12 and Alox15 in mouse wildtype. Quantitative RT-PCR of Alox15 in testis, epididymis and sperms of wildtype mice. Total RNA preparation and qRT-PCR was carried out as described in Materials and Methods. The Alox12 and Alox15 mRNA copy number/10^3 GAPDH mRNA copy number ratio was calculated to quantify the Alox12 and Alox15 expression levels.

4. Supplemental Figure S4. Impact of Alox15 deficiency on the extent of in vitro acrosome reaction.

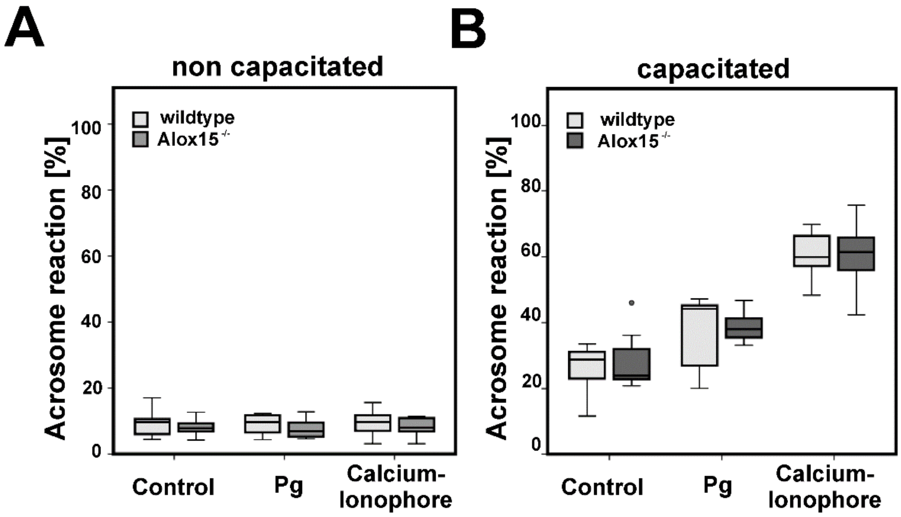

Figure S4. Alox15 deficiency does not affect the capability of the acrosome reaction. *In vitro* acrosome reaction. Epididymal cauda sperms were extracted from wildtype mice and Alox15$^{-/-}$ animals. The acrosome reaction was induced in non-capacitated (A) and capacitated (B) sperms with progesterone and A23187. The percentage of the acrosome reaction was quantified as described in Materials and Methods. Control indicates spontaneous acrosomal reaction in the DMSO solvent control sample. Significances were calculated using the Mann-Whitney U test. The experiment was carried out with 5-6 different individuals. At least 200-300 stained cells/ treatment were scored using Axioskop microscope (Zeiss, Germany) with a 40x objective clear field. Heads of sperms with the acrosome were stained dark blue (intact, not reacted acrosome) and without acrosome (unstained, reacted acrosome). The percentage of capacitated and non-capacitated acrosome reacted spermatozoa was calculated. *P*-values <0.05 were considered statistically significant.

5. Supplemental Figure S5. Analysis of oxygenated fatty acids in the lipid extracts of sperm of different genotype.

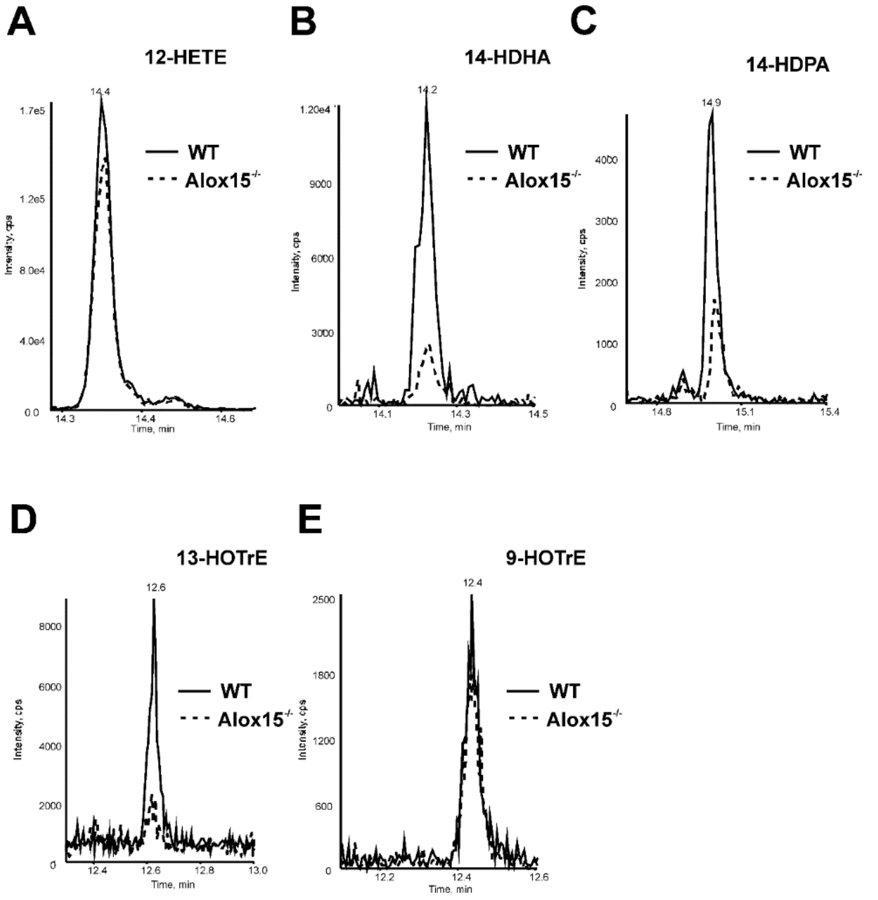

Figure S5. Representative chromatograms of Alox15 products in the lipid extract of sperms prepared from wildtype mice and *Alox15*-/- animals. Epididymal cauda sperms were prepared from wildtype mice and *Alox15*-deficient animals, total lipids extracted, hydrolyzed under alkaline conditions and selected hydroxy PUFAs analyzed by LC-MS/MS. Lipid extracts were analyzed by reverse-phase LC-MS/MS, on negative mode, using a Zorbax Eclipse Plus C18 column on 6500 Q Trap. *Panel A*. 12-hydroxy arachidonic acid (12-HETE); *Panel B*. 14-hydroxy docosahexaenoic acid (14-HDHA); *Panel C*. 14-hydroxy-docosapentaenoic acid (14-HDPA); *Panel D*. 13-hydroxy alpha linolenic acid (13-HOTrE); *Panel E*. 9-hydroxy alpha linoleic (9-HOTrE).

6. Supplemental Figure S6. Mass spectra of 14-HDPA found in the hydrolyzed lipid extracts of wildtype sperms.

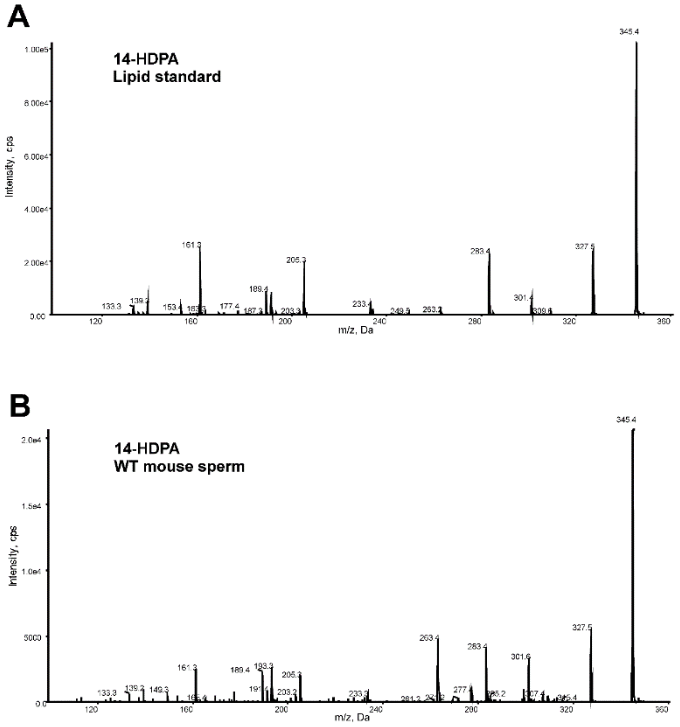

Figure S6. Comparative LC-MS/MS of 14-HDPA from wildtype mouse sperms and authentic standard. Epididymal cauda sperms were prepared from wildtype mice, total lipids extracted, hydrolyzed under alkaline conditions and selected hydroxy PUFA analyzed by LC-MS/MS. Lipid extracts were analyzed by reverse-phase LC-MS/MS, on negative mode, using a Zorbax Eclipse Plus C18 column on 6500 Q Trap. *Panel A-B.* MS/MS spectra of lipids detected as parent → 205.1 for 14-HDPA compared with synthetic standard. 14-HDPA standard was run under conditions identical to those used for WT mouse sperm lipid extracts. MS/MS spectra were acquired in ion trap mode at the apex of elution for each lipid. The spectrum is almost identical with the spectrum of the reference compound.

7. Supplemental Figure S7. Immune electron microscopy (immunogold staining) of wildtype mouse sperms indicating Gpx4 expression in different parts of the cell.

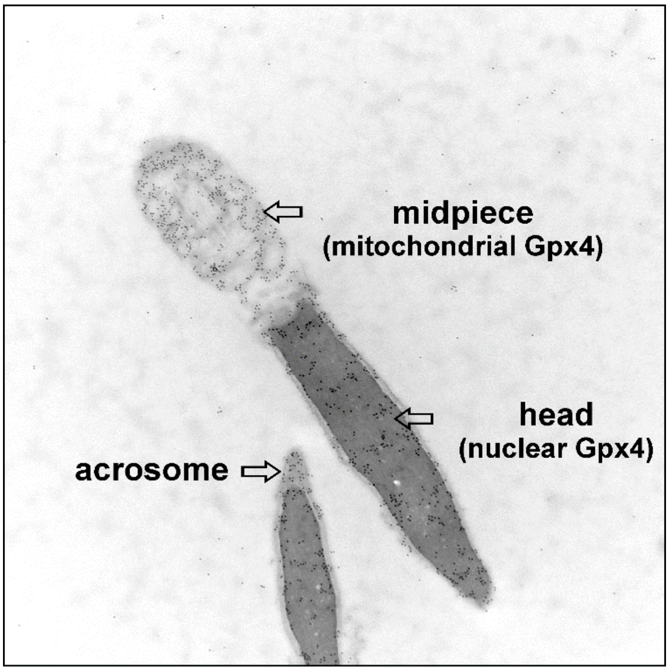

Figure S7. Immunogold staining of epididymal cauda sperm indicating the localization of Gpx4 in the head, midpiece and acrosome For immune electron microscopy sperm were fixed in Karnovsky's solution (Serva, Heidelberg, Germany) washed in PBS, dehydrated in ethanol and embedded in LR white resin. Ultrathin sections were incubated at room temperature with a 1:2000 dilution of our monoclonal antihuman Gpx4-antibody **[1]**. After one hour of incubation sections were washed with a few drops of PBS and then treated with a commercial goat anti-mouse IgG antibody (1:40 dilution), which was labeled with 12 nm colloidal gold. After 45 minutes at room temperature the sections were washed with PBS and water. Electron microscopy was carried out with FEI TecnaiSpiritBT device (120 kV; FEI Deutschland GmbH). The immunogold staining and imaging were performed by Karin Müller and Dagmar Viertel from Leibniz Institute for Zoo and Wildlife Research, Berlin, Germany.

From this image it can be seen that the midpiece the head and the acrosome were stained Gpx4 positive. This "unspecific-looking" staining pattern may not be related to low specificity of the used monoclonal Gpx4 antibody but rather to the fact that Gpx4 is present in all major parts of sperms. In fact, three major Gpx4 isoforms are encoded for by the Gpx4 gene and the corresponding proteins (cytosolic Gpx4, mitochondrial Gpx4, and nuclear Gpx4) are located in the different cellular compartments. Since the used antibody picks up all three Gpx4 isoforms, such unspecific looking expression patterns were actually expected.

8. Supplemental Figure S8. Immunoblotting of Gpx4-transfected HEK293 cells and corresponding mock-transfectants.

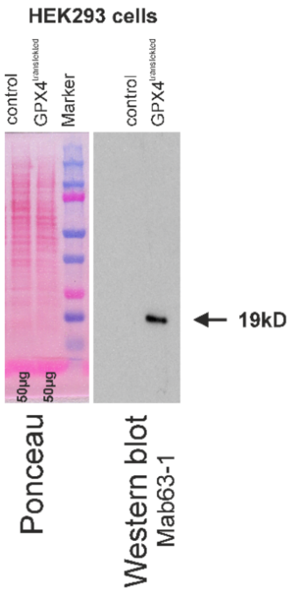

Figure S8. **Immunoblotting of Gpx4-transfected HEK293 cells and corresponding mock-transfectants.** HEK293 cells were transiently transfected with a pcDNA-based eucaryotic expression plasmid carrying the coding region of human Gpx4 cDNA. Mock-transfections were carried out with the empty expression plasmid. Cells were lysed by sonication and aliquots of the 20,000 g supernatants were applied to SDS-PAGE. After electrophoresis the proteins were blotted to a nitrocellulose membrane and the membrane was probed using our monoclonal anti-Gpx4-antibody **[1]**.

9. Gpx4 activity and protein expression in sperm cell lysates

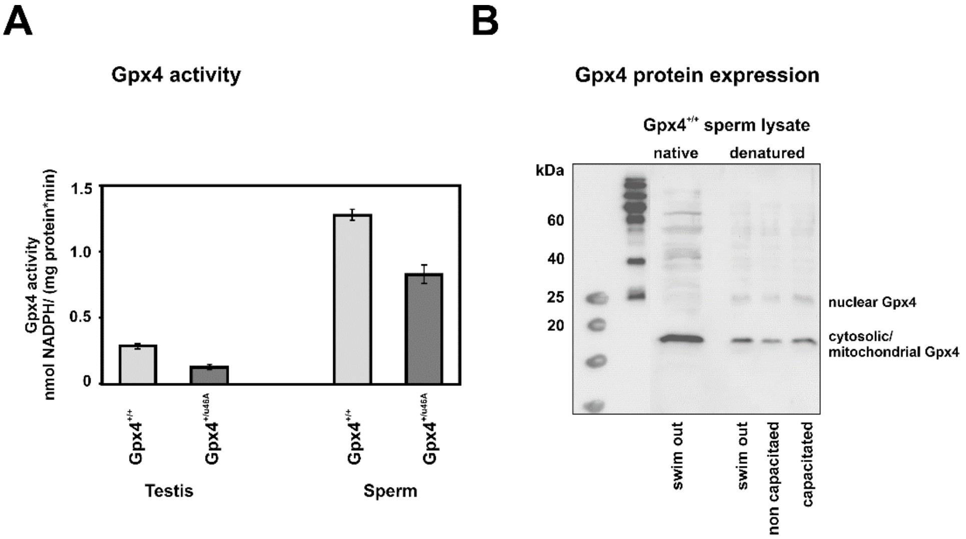

Figure S9. Gpx4 activity and Gpx4 protein expression in sperm. Epididymal cauda sperm were extracted from wildtype mice and Gpx4$^{+/U46A}$ animals as described in "Materials and Methods" .A) Gpx4 activity in isolated sperm. Sperm were freshly prepared and lysed in 0.4 ml of native lysis buffer (see Material and Methods). Gpx4 activity was assayed using the 10,000 g cell lysate supernatant as enzyme source and purified phosphatidylcholine hydroperoxide as substrate (see Material and Methods). For this experiment, the isolated sperm from three different mice of each genotype were pooled and the catalytic activity was measured in triplicate. When normalized to equal protein amounts in the sperm lysate supernatants we observed about 65% of the Gpx4 activity found in the lysate of wildtype sperm in the lysates of Gpx4$^{+/U46A}$ sperm. In the testis of Gpx4$^{+/U46A}$ mice 44% of the Gpx4 activity was measured compared with the wildtype mice. B) Expression of Gpx4 protein in sperm cells. Protein extracts were prepared from wildtype sperm lysate under native (25µg, lane "native") and denaturing conditions (about 200.000 sperm cells, lanes "denatured"). Aliquots of the protein extracts were applied to Western blotting using a monoclonal anti-human Gpx4 antibody (#MABF1969, Merck) **[1]** as described in Material and Methods. The Gpx4 protein in sperm was indicated by the immunoreactive band in the molecular weight range of about 18 kDa for the cytosolic and mitochondrial Gpx4 isoform or at about 25kDa for the nuclear Gpx4 isoform.

10. Immunohistochemical Gpx4 staining of wildtype mouse sperm cells

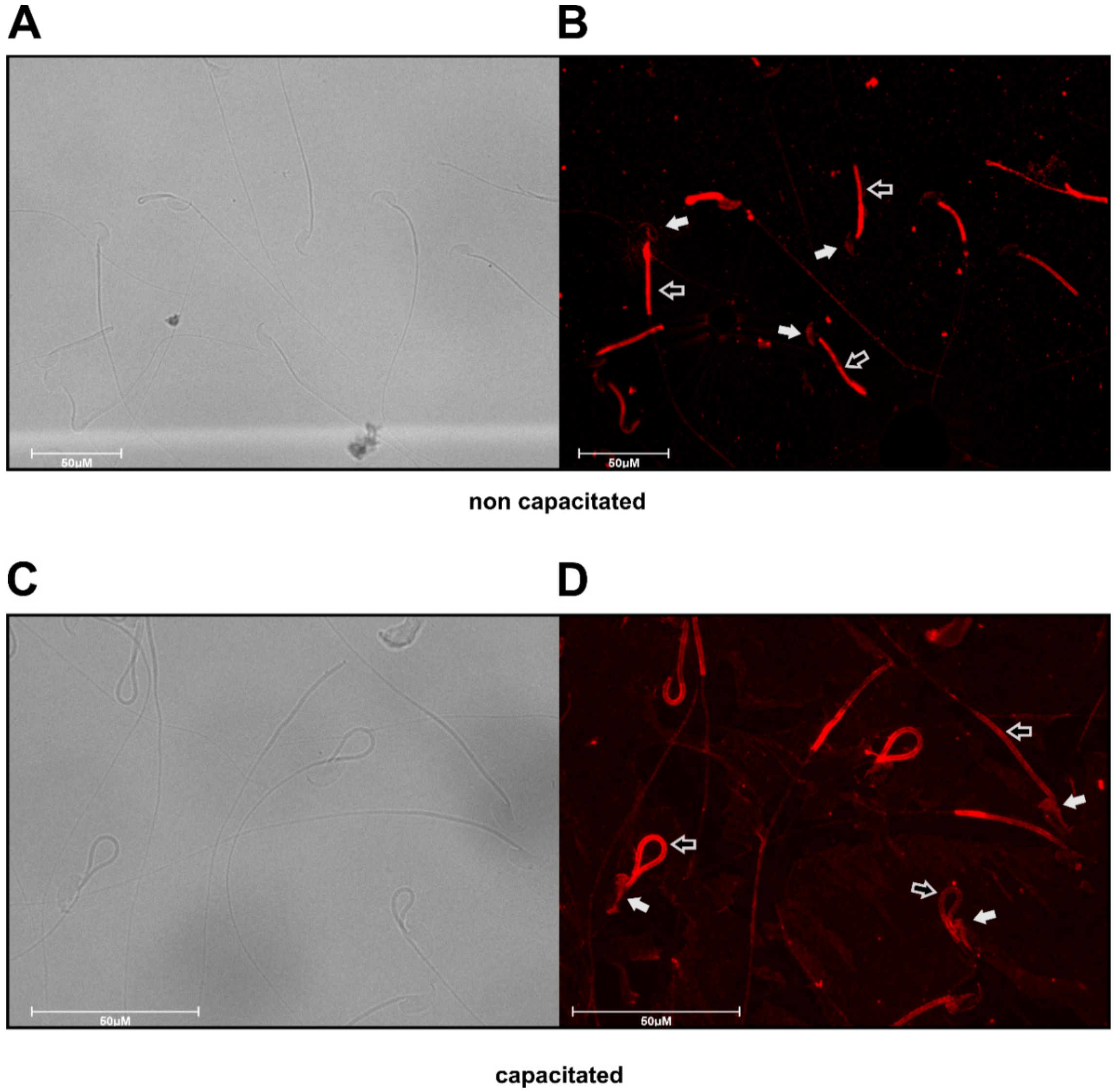

Figure S10. Immunohistochemical Gpx4 staining of wildtype mouse sperm before and after the capacitation. Sperm from epididymal cauda and vas deferens were extracted and capacitated. Immunohistochemical staining was carried out as described in the "Materials and Methods" section using the monoclonal anti-human GPX4 antibody (#MABF1969, Merck) **[1]**. A) Phase contrast visualization of non-capacitated sperm, B) Immunohistochemical Gpx4 staining of non-capacitated sperm, 60-fold. Midpiece (white framed arrows) and head (white arrows) were stained Gpx4 positive. C) Phase contrast visualization of capacitated sperm, D) Immunohistochemical Gpx4 staining of capacitated sperm, 100-fold. Midpiece (white framed arrows) and the post-acrosomal region (white arrows) were stained Gpx4 positive, whereas the anterior region of the sperm head was devoid of Gpx4.

1. Borchert, A.; Kuttner, G.; Giessmann, E.; Wang, C.C.; Wessner, H.; Volkmer, R.; Hohne, W.; Kuhn, H. Defining the immunoreactive epitope for the monoclonal anti-human glutathione peroxidase-4 antibody anti-hGPx4 Mab63-1. *Immunol Lett* **2010**, *133*, 85-93, doi:10.1016/j.imlet.2010.07.006
